# Characterization of *Pseudomonas aeruginosa* resistance to ceftolozane-tazobactam due to *ampC* and/or *ampD* mutations observed during treatment using semi-mechanistic PKPD modeling

**DOI:** 10.1128/aac.00480-23

**Published:** 2023-09-11

**Authors:** Luc Deroche, Vincent Aranzana-Climent, Albane Rozenholc, Laure Prouvensier, Léa Darnaud, Nicolas Grégoire, Sandrine Marchand, Marie-Cécile Ploy, Bruno François, William Couet, Olivier Barraud, Julien M. Buyck

**Affiliations:** 1 Université de Poitiers, PHAR2, Inserm U1070, Poitiers, France; 2 CHU de Poitiers, Département des agents infectieux, Poitiers, France; 3 Université de Limoges, Inserm U1092, Limoges, France; 4 CHU de Poitiers, Laboratoire de Toxicologie et de Pharmacocinétique, Poitiers, France; 5 CHU de Limoges, Laboratoire de Bactériologie-Virologie-Hygiène, Limoges, France; 6 CHU Limoges, Service de Réanimation Polyvalente, Limoges, France; 7 Inserm CIC 1435, CHU Limoges, Limoges, France; Shionogi Inc., Florham Park, New Jersey, USA

**Keywords:** *Pseudomonas aeruginosa*, ceftolozane/tazobactam, adaptive resistance, ampC, betalactamase, ampD, antibiotic resistance

## Abstract

A double *ampC* (AmpC^G183D^) and *ampD* (AmpD^H157Y^) genes mutations have been identified by whole genome sequencing in a *Pseudomonas aeruginosa* (PaS) that became resistant (PaR) in a patient treated by ceftolozane/tazobactam (C/T). To precisely characterize the respective contributions of these mutations on the decreased susceptibility to C/T and on the parallel increased susceptibility to imipenem (IMI), mutants were generated by homologous recombination in PAO1 reference strain (PAO1- AmpC^G183D^, PAO1-AmpD^H157Y^, PAO1-AmpC^G183D^/AmpD^H157Y^) and in PaR (PaR-AmpC^PaS^/AmpD^PaS^). Sequential time-kill curve experiments were conducted on all strains and analyzed by semi-mechanistic PKPD modeling. A PKPD model with adaptation successfully described the data, allowing discrimination between initial and time-related (adaptive resistance) effects of mutations. With PAO1 and mutant-derived strains, initial EC_50_ values increased by 1.4, 4.1, and 29-fold after AmpC^G183D^ *,* AmpD^H157Y^ and AmpC^G183D^/AmpD^H157Y^ mutations, respectively. EC_50_ values were increased by 320, 12.4, and 55-fold at the end of the 2 nd experiment. EC_50_ of PAO1-AmpC^G183D^/AmpD^H157Y^ was higher than that of single mutants at any time of the experiments. Within the PaR clinical background, reversal of AmpC^G183D^, and AmpD^H157Y^ mutations led to an important decrease of EC_50_ value, from 80.5 mg/L to 6.77 mg/L for PaR and PaR-AmpC^PaS^/AmpD^PaS^, respectively. The effect of mutations on IMI susceptibility mainly showed that the AmpC^G183D^ mutation prevented the emergence of adaptive resistance. The model successfully described the separate and combined effect of AmpC^G183D^ and AmpD^H157Y^ mutations against C/T and IMI, allowing discrimination and quantification of the initial and time-related effects of mutations. This method could be reproduced in clinical strains to decipher complex resistance mechanisms.

## INTRODUCTION

Multi-drug resistant (MDR) *Pseudomonas aeruginosa* resistant to carbapenems are among the critical priority pathogens list of WHO (1). β-lactams (BL) are the main therapeutic class used against these MDR bacteria, mostly associated with a β-lactamase inhibitor (BLI). Ceftolozane/tazobactam (C/T) is a BL/BLI with preserved efficacy on MDR *P. aeruginosa* (2, 3). Yet *P. aeruginosa* resistance to C/T has been observed in patients on several occasions (4 – 11), mainly due to mutations in *ampC* gene associated with mutations in *ampC* regulators, such as *ampR* and/or *ampD* (4, 8, 11). However, the identification of a particular mutation hardly predicts the precise reduction of antibiotic susceptibility especially since the same *ampC* mutation can induce increased resistance to C/T but restore the susceptibility to imipenem (IMI) (12). Furthermore *P. aeruginosa* resistance development may arise from multiple factors [mutations in chromosomal resistance gene(s), single nucleotide polymorphisms or deletions, horizontal acquisitions of resistance gene(s)] (13), and take several forms (acquired or adaptive resistance) that cannot be discriminated by minimum inhibitory concentration (MIC) determinations (14). To cope with that issue, semi-mechanistic PKPD modeling approaches have been developed to consider the full time-course of bacterial growth, killing, and emergence of resistance in response to different antibiotic exposure profiles (15).

We have detected a resistance to C/T that appeared during treatment in a clinical isolate of *P. aeruginosa*, after mutations in *ampC* and *ampD*. Thus, the objective of this study was to characterize the decreased susceptibility to C/T induced by those mutations, using *P. aeruginosa* PAO1 reference and mutant-derived strains as well as semi-mechanistic PKPD modeling, to capture the effect of each mutation on acquired and adaptive resistance. In parallel, the developed approach has been used to characterize the effect of these mutations on IMI susceptibility recovery.

## RESULTS

### Characterization of clinical isolates and mutant-derived strains

The whole genome sequencing (WGS) analysis confirmed that the MDR IMI-resistant *P. aeruginosa*, susceptible to C/T (PaS) and the C/T resistant but IMI susceptible *P. aeruginosa* (PaR) clinical isolates belonged to the same sequence type ST252 and were isogenic. Analysis of Single Nucleotide Polymorphisms (SNPs) has shown around 300 SNPs in PaR compared to PaS (Table S1). This led to the identification of two mutations with possible implication in resistance to C/T: a C548A nucleotide substitution, leading to a G183D substitution in the chromosomal cephalosporinase (AmpC), and a C469T nucleotide mutation, leading to a H157Y substitution in AmpD, an N-acetyl-anhydromuramyl-L-alanine-amidase that negatively controls expression of *ampC* gene.

To decipher the role of each mutation on C/T resistance, two PAO1 strains with single mutation (PAO1-AmpC^G183D^ and PAO1-AmpD^H157Y^) and one PAO1 strain with double mutations (PAO1-AmpC^G183D^/AmpD^H157Y^) were obtained. An increase in basal *ampC* expression was observed in the isogenic PAO1 mutant strain carrying the AmpD^H157Y^ mutation (Fig. S1).

C/T MIC values of PAO1-AmpC^G183D^ and PAO1-AmpD^H157Y^ were identical to that of PAO1 (MIC = 0.25 mg/L), while that of the PAO1 double mutant (PAO1-AmpC^G183D^/AmpD^H157Y^) was increased eightfold (MIC = 2 mg/L). A double reverted PaR isolate (PaR-AmpC^PaS^/AmpD^PaS^) was obtained with a MIC close to that of PaS (MIC = 1 mg/L) and considerably reduced compared with that of PaR (MIC = 2 mg/L versus 32 mg/L) (Table 1).

**TABLE 1:** Minimal inhibitory concentrations (mg/L) and EC_50_ of C/T at the start (EC_50,off_) of the first TKC and at the end (EC_50,on_) of the second TKC (mg/L) of PAO1 and mutant-derived strains and of clinical pair of *P. aeruginosa* (PaS and PaR), and isogenic mutant-derived PaR^*a*^

	MIC	EC_50,off_ [CI 95%]^*c*^	EC_50,on_ [CI 95%]	EC_50,on_/EC_50,off_
	(mg/L)	mg/L	mg/L	
Mutant-derived reference strains				
PAO1	0.25	0.0444 [0.00818–0.104]	0.551 [0.408–0.743]	12.4
PAO1- AmpC^G183D^	0.25	0.0606 [0.0294–0.0988] (1.4^*b*^)	19.4 [15.8–23.4]	320
PAO1- AmpD^H157Y^	0.25	0.181 [0.121–0.263] (4.1^*b*^)	2.20 [1.83–2.59]	12.2
PAO1- AmpC^G183D^/AmpD^H157Y^	2	1.29 [0.499–2.55] (29^*b*^)	70.8 [60.5–83.1]	55
Mutant-derived clinical isolates				
PaS	1	1.36 [1.04–1.73]	155 [124–92]	114
PaR	**32^*d*^**	*80.5* *[66.4–92]* (59^*b*^)	420 [371–485]	*5.2*
PaR-AmpC^PaS^/AmpD^PaS^	2	*6.77* *[5.37–8.17]* (5.0^*b*^)	269 [220–345]	*40*

^*a*^ Results in *italics* should be interpreted with caution, as the PaR strain was previously exposed to C/T, thus could be already adapted at the start of the first TKC.

^*b*^ fold-change compared to initial strain (PAO1 or PaS).

^*c*^ CI 95%, Confidence intervals 95%.

^*d*^ In Bold: resistant considering EUCAST Breakpoint of 4 mg/L.

Concerning IMI, the clinical isolate PaS was resistant to IMI with a MIC of 4 mg/L, whereas the clinical isolate PaR was susceptible with a MIC value of 1 mg/L. MICs of PAO1 and PAO1-AmpD^H157Y^ to IMI were 0.5 mg/L. The values of MIC for strains carrying the AmpC^G183D^ mutation (PAO1-AmpC^G183D^ and PAO1-AmpC^G183D^/AmpD^H157Y^) were decreased to 0.125 mg/L (Table 2).

**TABLE 2:** Minimal inhibitory concentrations (mg/L) and EC_50_ of IMI at the start (EC_50,off_) of the first TKC and at the end (EC_50,on_) of the second TKC (mg/L) of PAO1 and mutant-derived strains

	MIC	EC_50,off_ [CI 95%]^*b*^	EC_50,on_ [CI 95%]	EC_50,on_/EC_50,off_
	(mg/L)	mg/L	mg/L	
Mutant-derived reference strains				
PAO1	0.5	0.411 [0.250–0.638]	24.8 [21.3–29.3]	60.3
PAO1- AmpC^G183D^	0.125	1.86 [1.49–2.26] (4.5^*a*^)	2.25 [1.91–2.57]	1.2
PAO1- AmpD^H157Y^	0.5	2.03 [1.58–2.64] (4.9^*a*^)	7.68 [4.62–11.1]	3.8
PAO1- AmpC^G183D^/AmpD^H157Y^	0.125	0.495 [0.397–0.603] (1.2^*a*^)	3.29 [2.84–3.80]	6.6

^*a*^ fold-change compared to initial strain PAO1.

^*b*^ CI 95%, Confidence intervals 95%.

### PKPD modeling

No degradation of C/T was observed in MHB. IMI degradation followed a mono-exponential decay with degradation half-life t_1/2_= 16.4 h (data not shown) comparable with 17 hr which was found by Yadav *et al*. in the same experimental conditions (16).

**Fig 1:**
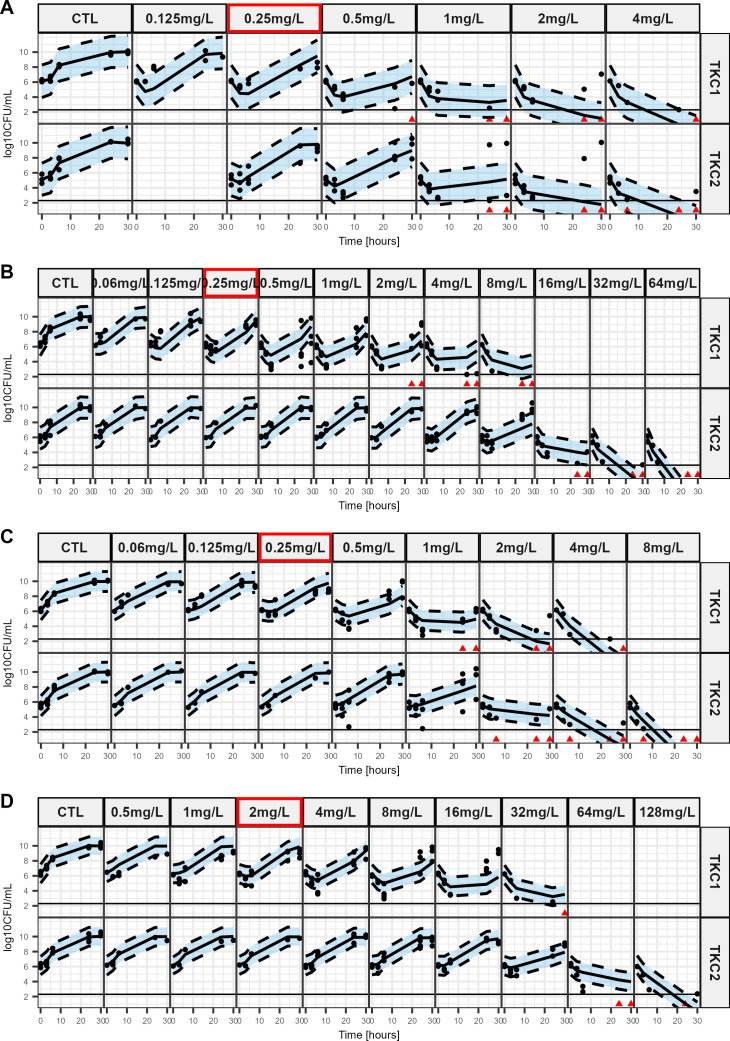
Visual predictive checks of the final model based on *in vitro* sequential time kill data of ceftolozane/tazobactam for PAO1 and mutant-derived strains. The plots show the observed time-kill data at different C/T concentrations with model predictions as medians and 80% confidence intervals around the median (shaded area). The red frame represents the MIC value of the corresponding strain. Dots represent countable plates; red triangles represent data below the limit of quantification (BLQ: 2.3 log10 CFU/mL). Data below the limit of detection are plotted as 0.1 CFU/mL. (A). PAO1 (B). PAO1-AmpC^G183D^, (C). PAO1- AmpD^H157Y^, (D). PAO1-AmpC^G183D^/AmpD^H157Y^.

C/T experimental data are represented as dots in Fig. 1 for PAO1 mutant-derived strains and on Fig. S2 for clinical isolates. For PAO1 mutant-derived strains and clinical isolates, the first time-kill curves (TKCs) showed bactericidal effect of C/T for concentrations over their corresponding MIC. Initial CFU decreased followed by a regrowth occurred for all mutant strains until the concentration corresponding to MIC in the first time-kill but for higher concentration in the second time-kill, particularly for PAO1-AmpC^G183D^ (Fig. 1B) and the double mutant (Fig. 1D).

For all strains, killing and regrowth were well described by the model (Fig. 1; Fig. S2), and susceptibilities to C/T are reflected by EC_50_ values before (EC_50,off_, 2nd column) and after (EC_50,on_, 3rd column) exposure to C/T (Table 1).

For PAO1 mutant-derived strains, AmpD^H157Y^ mutation induced acquired resistance, with a 4.1-fold increase in EC_50,off_ compared to PAO1 (Table 1), while the AmpC^G183D^ mutation had a similar EC_50,off_ than PAO1 with a 1.4-fold increase and an overlapping 95% confidence interval (CI). But AmpC^G183D^/AmpD^H157Y^ double mutation had the greatest effect with an EC_50,off_ value increased by 29-fold. In terms of adaptive resistance, PAO1 EC_50,on_ was 12.4-fold higher than its corresponding EC_50,off_. This ratio between EC_50,on_ and EC_50,off_ was virtually unchanged (12.2) after AmpD^H157Y^ mutation and tremendously increased (320-fold) after AmpC^G183D^ mutation, but intermediate (55) after AmpC^G183D^/AmpD^H157Y^ double mutation.

For clinical isolates, EC_50,off_ value increased from 1.36 mg/L for PaS to 80.5 mg/L for PaR corresponding to a 59-fold difference and decreased to 6.77 mg/L for the double reverted PaR mutant. PaS EC_50,on_ was estimated at 155 mg/L, that is 114-fold higher than EC_50,off_.

Other modeling parameters values for C/T are provided in Table S2 and S3.

**Fig 2:**
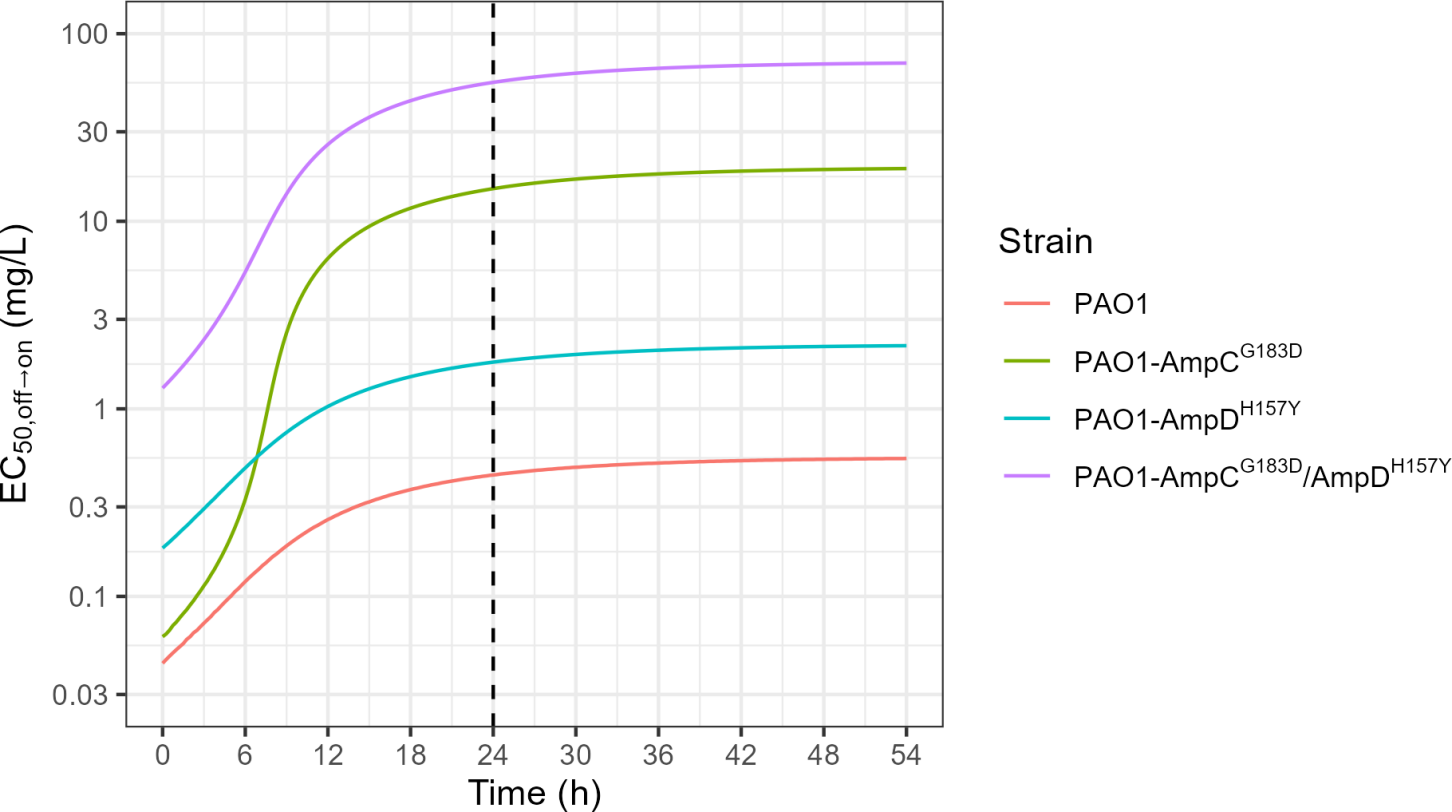
EC_50,off→on_ over time simulated from the final model based on the C/T *in vitro* sequential time-kill data. Time is defined as the time since the beginning of the first time-kill. Each colored line shows the empirical EC_50_ for a different strain. Dashed vertical line shows time = 24 hours which is the time at which samples from the first time-kill are taken to prepare the inoculum of the second time-kill.

The effect of time on PAO1 and mutant-derived strains susceptibility to C/T (EC_50_, _off→on_), reflecting adaptive resistance development, is illustrated in Fig. 2. Simulations showed that at any time, for C/T, EC_50_, _off→on_ of PAO1 is always the lowest, whereas that of PAO1-AmpC^G183D^/AmpD^H157Y^ is always the highest. The PAO1-AmpC^G183D^ curve starts at almost the same value than that of PAO1 and increase rapidly from 6 hours until to reach a plateau. The PAO1-AmpD^H157Y^ curve evolves parallel to that of double mutant strain.

**Fig 3:**
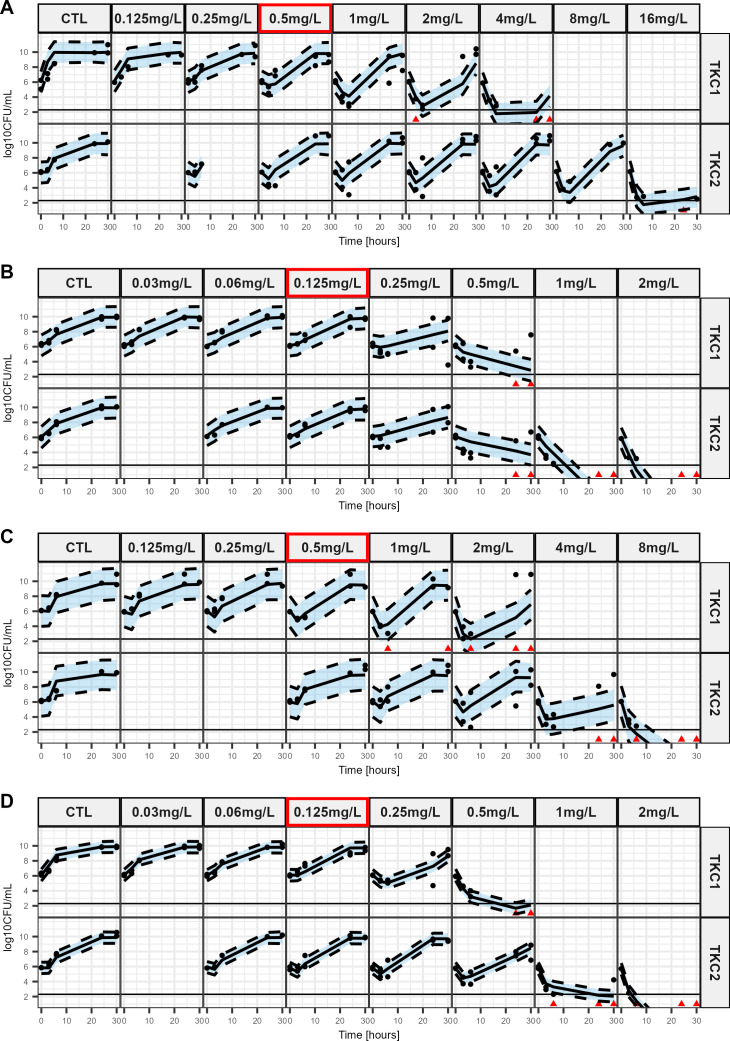
Visual predictive checks of the final model based on *in vitro* sequential time-kill data of imipenem for PAO1 and mutant-derived strains. The plots show the observed time-kill data at different IMI concentrations with model predictions as medians and 80% confidence intervals around the median (shaded area). The red frame represents the MIC value of the corresponding strain. Dots represent countable plates; red triangles represent data below the limit of quantification (BLQ: 2.3 log10 CFU/mL). Data below the limit of detection are plotted as 0.1 CFU/mL. (A) PAO1 (B) PAO1-AmpC^G183D^, (C) PAO1- AmpD^H157Y^, (D) PAO1-AmpC^G183D^/AmpD^H157Y^.

IMI experimental data for PAO1 and mutant-derived strains are represented as dots in Fig. 3. For all strains, initial CFU decrease followed by a regrowth occurred in the first time-kill for concentration close to (PAO1-AmpC^G183D^, Fig. 3B and the double mutant, Fig. 3D) or slightly higher (PAO1, Fig. 3A and PAO1-AmpD^H157Y^, Fig. 3C) than the corresponding MIC. In the second time-kill, regrowths at higher concentrations are mainly observed for PAO1. Killing and regrowth were well described by the model (Fig. 3), and susceptibilities to IMI are reflected by EC_50_ values before (EC_50,off_, 2nd column) and after (EC_50,on_, 3rd column) exposure to IMI (Table 2).

For PAO1 and mutant-derived strains, AmpC^G183D^ and AmpD^H157Y^ mutations alone induced greater acquired resistance than AmpC^G183D^/AmpD^H157Y^ double mutation, with respectively a 4.5, 4.9, and 1.2-fold (with an overlap in 95% CI) increase in EC_50,off_ compared to PAO1 (Table 2). In terms of adaptive resistance, PAO1 EC_50,on_ was 60.3-fold higher than its corresponding EC_50,off_ while it increased a lot less for the mutant-derived strains. Notably, strains with the AmpC^G183D^ mutation have markedly lower EC_50,on_ than PAO1 (2.25 and 3.29 mg/L vs 24.8 mg/L) and slightly lower than PAO1-AmpD^H157Y^ (2.25 and 3.29 mg/L vs 7.68 mg/L). Other model parameters values for IMI are provided in Table S4.

**Fig 4:**
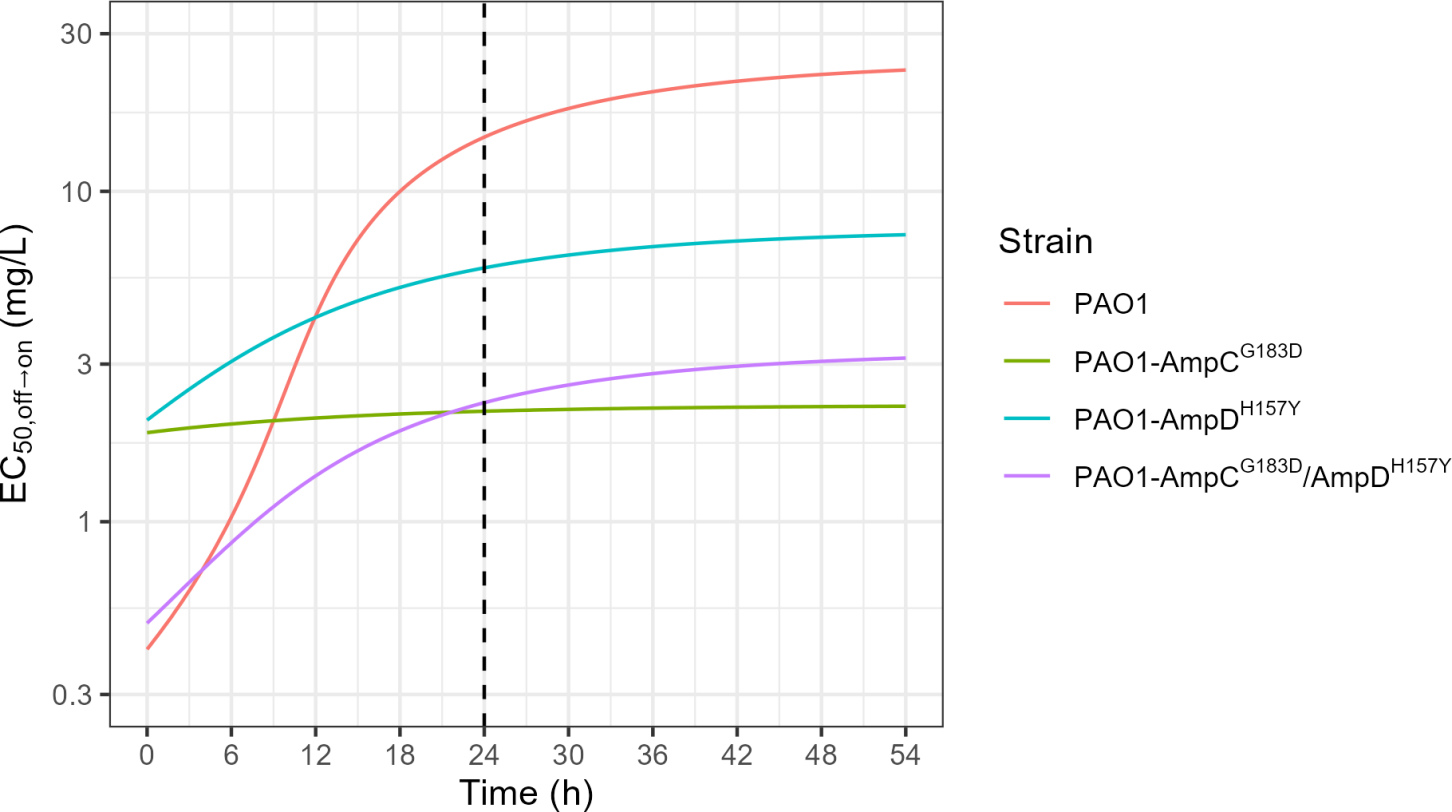
EC_50,off→on_ over time simulated from the final model based on the imipenem *in vitro* sequential time-kill data. Time is defined as the time since the beginning of the first time-kill. Each colored line shows the empirical EC_50_ for a different strain. Dashed vertical line shows time = 24 hours which is the time at which samples from the first time-kill are taken to prepare the inoculum of the second time-kill.

The effect of time on PAO1 and mutant-derived strains susceptibility to IMI, reflecting adaptive resistance development, is illustrated in Fig. 4. Simulations showed that the PAO1 curve increased with time to reach the highest EC_50,off→on_ value at 54 hours. PAO1-AmpC^G183D^ curve is almost flat and the PAO1-AmpD^H157Y^ and the PAO1-AmpC^G183D^/AmpD^H157Y^ double mutant curves are almost parallel.

Moreover, the EC_50, off→on_ at 24 hours of PAO1 and mutant-derived strains were consistent with MIC values (Fig. 4 and Table 2) with higher EC_50, off→on_ for PAO1 and PAO1-AmpD^H157Y^ than for PAO1-AmpC^G183D^ and the PAO1-AmpC^G183D^/AmpD^H157Y^ double mutant strain.

## DISCUSSION

AmpC^G183D^ and AmpD^H157Y^ mutations detected in PaR, and presumably responsible for C/T resistance associated with recovery of IMI susceptibility, were reproduced in a reference PAO1 strain to investigate specifically their consequences on C/T and IMI resistance variability. Mutants were generated using homologous recombination, which has the advantages over other commonly used methods, of being scarless and reflecting the gene function in its native context (17). Semi-mechanistic PKPD modeling was conducted from sequential TKC data to facilitate the selection of a model (18), and allow precise quantitative discrimination between acquired resistance due to mutation and consecutive adaptive resistance (19, 20).

AmpC^G183D^ mutation induced limited, if any, acquired resistance of PAO1 to C/T (EC_50,off_ increased by only 1.4-fold). This mutation was first described *in vitro* (4) and later *in vivo* after treatment by C/T (8, 21, 22). Its effect on C/T resistance has been investigated by using an expression plasmid harboring AmpC^G183D^ in a PAO1 reference strain, knocked-out for *ampC* (4), and reporting high-level resistance to C/T (MIC = 32 mg/L). Yet pUC plasmids are high-copy number plasmids, leading to an overexpression of the plasmid genes (23), whereas homologous recombination, which has been used in this study, allows the determination of the effect of AmpC^G183D^ in the native context. Other authors have cloned the *ampC* gene with its promoter into *Escherichia coli* to investigate the effect of the AmpC^G183D^ substitution, confirming its role in C/T resistance (8). A recent study (24) showing that this mutation allows more flexibility to AmpC leading to an enhanced enzyme affinity toward ceftolozane could explain the adaptive resistance to C/T observed in the PAO1-AmpC^G183D^ strain (Table 1).

Mutations in AmpD are expected to increase *ampC* expression, leading to high β-lactamase production (25, 26). Indeed, AmpD^H157Y^ mutation in this study induced a basal overexpression of *ampC*, confirmed by qPCR experiments on PAO1 mutant-derived strains (Fig. S1). AmpD^H157Y^ mutation was responsible for acquired resistance of PAO1 to C/T, characterized by a 4.1-fold increase of EC_50,off_. In the present study, initial higher-level expression of *ampC* may be responsible for a limited initial decrease in susceptibility to C/T. However, this *ampC* overexpression had no effect on adaptive resistance, as EC_50,on_/EC_50,off_ ratios were similar between PAO1 and PAO1-AmpD^H157Y^ (Table 1).

More interestingly, the double mutations led to a “synergistic type” of effect on the acquired resistance to C/T, with an EC_50,off_ value for PAO1-AmpC^G183D^/AmpD^H157Y^, 29-fold higher than that of PAO1 (1.29 mg/L vs 0.0444 mg/L, respectively). The observation was different for adaptive resistance, which was moderate for the AmpD^H157Y^ mutant (EC_50,on_/EC_50,off_ = 12) and much higher for the AmpC^G183D^ (EC_50,on_/EC_50,off_ = 320), but intermediate for PAO1-AmpC^G183D^/AmpD^H157Y^ (EC_50,on_/EC_50,off_ = 55). However, the difference in susceptibility to C/T between the PAO1-AmpC^G183D^/AmpD^H157Y^ and the wild-type PAO1 increased with adaptation as shown by a 128-fold increase of EC_50,on_ (70.8 mg/L and 0.551 mg/L, respectively) compared with 29-fold increase for the EC_50,off_ values. These results highlight previous observations suggesting that high-level C/T resistance was achieved when mutations occurred on both *ampC* gene and *ampC* regulators (4).

The combined effects of acquired and adaptive resistance on C/T with time, induced by the various mutations are illustrated in Fig. 2. The PKPD model with adaptation used to describe these experimental data has been initially proposed by Mouton et al. and since that, used on a number of occasions (15, 27, 28). It assumes that adaptation starts at time zero (when 100% of the bacteria are in the “off” state), and keeps developing until the bacterial population becomes fully adapted (100% “on”). Yet, the adaptation development rate constitutes an interesting but neglected information. This issue is complexified here by the presence of two mutations. During adaptation development, when only a continuously changing fraction of the bacterial population is adapted, the bactericidal effect is no more characterized by an E_max_ model, but by an equation combining two E_max_ models, one for the “on” and the other for the “off” bacteria (Eq 1). For any given fraction of the bacterial population that is adapted, we could determine the C/T concentration at which 50% of this maximum effect is reached, defined as EC_50,off→on_. However, as opposed to the EC_50_ value of an E_max_ model, this parameter value is not fixed but varies with the fraction of the bacterial population that is adapted (Fig. S3a), which itself varies with time (Fig. S3b). Because of these two driving effects, hence EC_50,off→on_ is time-dependent (Fig. S3c).

In simulations (Fig. 2), The PAO1 and PAO1-AmpD^H157Y^ curves are parallel, consistent with the fact that AmpD^H157Y^ mutation confers initial resistance but then similar adaptive resistance to PAO1. The PAO1-AmpC^G183D^ curve starts at almost the same value than that of PAO1 due to the virtual lack of acquired resistance, but raises very sharply to cross the PAO1-AmpD^H157Y^ curve after about 6 hours. Figure 2 also shows that adaptive resistance develops rapidly in these *in vitro* conditions, with about 90% of adaptation reached after 24 hours.

In the clinical isolates, only the double mutant was identified after treatment. Yet, results obtained with PaS and PaR are more difficult to interpret than those obtained with PAO1 and mutant-derived strains, since the two types of resistance cannot be differentiated in PaR. Acquired resistance was characterized genotypically, whereas adaptive resistance, likely due to multiple and complex variations of gene expression, could only be characterized indirectly and unspecifically from reduction of antimicrobial efficacy assessed by EC_50_ determinations. Indeed, PaS had never been exposed to C/T, whereas PaR was recovered after a 2 weeks C/T treatment. Therefore, adaptive resistance may have developed *in vivo* in PaR. Then, comparing PaR and PaS, EC_50,off_ values (80.5 and 1.36 mg/L) may reflect a mixed effect due to acquired and adaptive resistance. Yet, the relatively low EC_50,off_ value of PaR-AmpC^PaS^/AmpD^PaS^ (6.77 mg/L), almost back to that of PaS (1.36 mg/L), suggests that this double mutation explains most but not all of PaR resistance to C/T.

In this clinical case, as PaS became PaR during treatment, C/T resistance increased (32-fold MIC increase), while IMI resistance reverted (eightfold MIC reduction). This reversion could be linked with AmpC^G183D^ mutation that has been found in PaR, since it has been previously described to decrease the MIC of IMI (4, 8, 12, 22, 24). This observation was confirmed in our study by a fourfold IMI MIC decrease of PAO1 mutant-derived strains containing the AmpC^G183D^ mutation (Table 2). Simulations (Fig. 4 and Table 2) showed that strains carrying the AmpC^G183D^ mutation are less prone to adaptive resistance with flattened curves and lower EC_50,off→on_ from 24 hours (Fig. 4) compared to strains with a wild-type AmpC. This is consistent with previous studies that have shown AmpC mutations (including AmpC^G183D^) decrease the affinity to imipenem (4, 8, 12, 22, 24). Regarding the AmpD^H157Y^ mutation, the effect appeared to be less important than AmpC^G183D^ mutations but slows adaptive resistance over time compared to PAO1 illustrated by lower EC_50,off→on_ from the 12th hour (Fig. 4). However, EC_50,off→on_ at time 0 is more difficult to interpret since the strains with single mutations presented a higher EC_50,off→on_ value than PAO1 wild type and PAO1 carrying the double mutation.

Several limitations of the study should be noted. Most of the information provided by this study come from PAO1 and derived strains, which constitutes an optimal tool for precise mechanistic characterizations, but do not represent clinical isolates. Furthermore, data obtained *in vitro* should always be very carefully extrapolated in clinics. In this study, we focused on the two clinically observed mutations that could be directly related with known mechanisms of resistance to ceftolozane, AmpC^G183D^ and AmpD^H157Y^. However, while the double reverted mutant PaR-AmpC^PaS^/AmpD^PaS^ has showed decreased C/T MIC and EC_50,off_, it was still less susceptible to C/T than PaS. Thus, even if their roles are probably minor, the effect of other mutations among the SNPs observed in PaR compared to PaS cannot be excluded. Likewise, the other mutations present in the genes involved in the regulation or in *ampC* of the clinical isolates that appeared before C/T treatment were not reproduced in PAO1. For example, both clinical isolates (PaS and PaR) had two other mutations in *ampC* gene (AmpC^T105A^ and AmpC^R233D^) and one mutation in the AmpC-repressor AmpR (AmpR^G85S^) in comparison with PAO1. These mutations could have a cumulative effect with the AmpC^G183D^ and AmpD^H157Y^ mutations identified in the PaR strain after C/T treatment.

PAO1 strains and their mutants exhibited different growth patterns in the C/T and the IMI experiments. Indeed, even in absence of drug a lag-time could be present in one set of experiments but not in the other as evidenced by the T_lag_ parameter estimates in Table S2 and S4 and the growth curves in Fig. 1 and 3. We could not find and experimental or biological reason for this intriguing phenomenon. However, the goal of the study was to quantify the *in vitro* difference of effects between the different studied strains rather than mechanistically describe to bacterial growth. Thus, we decided to estimate growth parameters specific to each experiment set, leading to a proper yet overfitted description of the growth data. This enabled us to have a correct quantitative description of drug effect while having the adverse effect of making the model unsuitable for predictions of future experiments before any external validation is conducted.

Another limitation of the study is that due to the limited number (*n* = 3) of replicates for each drug-bug pair, the observed variability in bacterial response could not be attributed to a specific model parameter but only quantified through the residual unexplained variability term (σ2, Table S2and S4). Notably, this variability parameter is high for C/T against PAO1 (Table S2, 1st column) and C/T against PAO1-AmpD^H157Y^ (Table S4, 3rd column). These high variabilities, reflect the presence of concentrations for which regrowth was observed in some replicates but not in others, e.g., 1 and 2 mg/L for the second TKC of C/T against PAO1 (Fig. 1A). Again, this limits the predictive ability of the model while not invalidating the quantitative description of the experimental data.

In this study, we showed that AmpC^G183D^ AmpD^H157Y^ double mutation in *P. aeruginosa* was responsible for acquisition of resistance to C/T in a clinical and a laboratory strain. In the same strains, we also showed that AmpC^G183D^ was mainly responsible for reversal of resistance to IMI. Using newly created PAO1 mutants, sequential time-kill curves and PKPD modeling, we were able to precisely quantify the impact of each mutation on acquired and adaptive resistances to C/T and IMI.

## MATERIALS AND METHODS

### Patient characteristics and clinical isolates

A 58-year-old female patient, under chemotherapy for breast cancer, developed a catheter-related bacteremia. Empirical treatment (IMI 1 g tid plus amikacin 30 mg/kg qd) was started, then replaced 2 days later by C/T (Zerbaxa, MSD, Puteaux, France), 1.5 g tid, after an MDR IMI-resistant *P. aeruginosa* (MIC of 8 mg/L), susceptible to C/T (PaS) with an MIC equal to 1 mg/L, was isolated. After 2 weeks of treatment, a C/T resistant but IMI susceptible *P. aeruginosa* (PaR) was isolated from blood cultures with MIC equal to 32 mg/L and 1 mg/L for C/T and IMI, respectively. Patient was eventually treated successfully with imipenem/cilastatin 0.5 g qid for 14 days.

Whole genome sequence analysis of PaS and PaR was conducted by Ion Proton technology (Thermo Fisher Scientific, Waltham, MA, USA) according to the manufacturer’s instructions. Quality check of raw reads was done using FastQC v.0.11.5 and MultiQC v.0.9, and no trimming was performed. Mean coverage of 40× was determined after alignment against the *P. aeruginosa* PAO1 reference genome (GenBank GCA_000006765.1). Reads were then assembled using Mimicking Intelligent Read Assembly software. Contigs were analyzed using Geneious software (Biomatters Ltd., Auckland, New Zealand). Antimicrobial resistance genes were identified using ResFinder 4.0 (29). Genes with a 60% minimum length and a percentage of identity >98% were considered. Sequence types (STs) were determined using the *Pseudomonas* multilocus sequence typing database (https://cge.cbs.dtu.dk/services/MLST/). SNPs were called against PAO1 reference sequence (GenBank GCA_000006765.1) using freebayes (v.1.3.1) (30).

### Construction of *ampC*/*ampD* mutant strains by homologous recombination and characterization

All the strains and primers used in the study are presented in Tables S5 and S6. *P aeruginosa* PAO1 (ATCC 15692) was used as a reference strain for mutant production. Construction of plasmids and induction of AmpC and AmpD mutations was accomplished as previously described (31), with slight modifications. Briefly, 700 base pair sequences of the flanking regions of the targeted mutation were PCR-amplified with primers containing the mutation. All primers were designed with Snapgene software (from Insightful Science; available at snapgene.com). The PCR fragments were gel-purified and inserted into pEXG2-linearized plasmid by Gibson assembly (New England Biolabs, Evry, France). The assembled plasmid was transformed into competent *E. coli* JKe201 and then plated on Luria Bertani (LB) agar containing 10 mg/L of gentamicin and 100 µM of diaminopimelic acid. Sequenced-verified clones were mated for 4 hours with the PAO1 reference strain at 37°C and then plated on LB agar containing 30 mg/L of gentamicin allowing to select PAO1 that have integrated the plasmid. Colonies were picked and grown in LB for 4 hours and streaked on 20% sucrose plates overnight at 30°C. Targeted mutations in clones were confirmed by Sanger sequencing.

For clinical strains, AmpC^G183D^ and AmpD^H157Y^ mutations found in PaR isolate were reverted by cloning part of the whole *ampC* and *ampD* genes from PaS into PaR, using a similar protocol. Seven hundred base pair sequences upstream and downstream of the targeted mutation in *ampC* or *ampD* genes were PCR-amplified from the susceptible PaS clinical isolate and then assembled by Gibson assembly into pFOG plasmid (17). Then, the mating step was done with *E. coli* JKE201 containing pFOG plasmid and PaR resistant isolate. Sanger sequencing was used to confirm the presence of the mutation in each mutant strain.

### *ampC* gene expression analysis

Bacterial suspensions were collected to obtain a maximum inoculum of 10^9^ CFU. Samples were centrifuged (12,000 g, 5 minutes), and the pellets were extracted with the RNeasy Mini Kit (Qiagen) according to the manufacturer’s instructions. Bacteria were lysed using 0.1 mm Glass Beads (Scientific industries) for 15 minutes under horizontal shaking. Samples were treated with the TURBO DNA-Free Kit using the rigorous protocol (Thermo Fischer Scientific, Illkirch-Graffenstaden, France) to eliminate residual DNA. The concentration of total RNA was estimated using the Nanodrop One system (Thermo Fischer Scientific, Illkirch-Graffenstaden, France).

Extracted total RNA was used to synthetize cDNA with the FIREScript RT cDNA synthesis kit (Solis Biodyne, Tartu, Estonia) according to the manufacturer’s instructions, using an iCycler PCR system (Bio-Rad, Marnes-la-Coquette, France).

Primers were designed with Geneious Prime software (Biomatters Ltd., Auckland, New Zealand). Quantitative PCR was performed from cDNA with the ONEGreen FAST qPCR Premix (Ozyme, Saint-Cyr-l'École, France) using the CFX96 Touch Real-Time PCR Detection System. All samples were analyzed in duplicate, and the relative expression of genes was normalized by the expression of a reference gene (*rpsl*). The relative differences in mRNA expression levels were determined using comparative cycle threshold (Ct) method (2^-ΔΔCt^). The results were analyzed with the CFX Maestro Software (Bio-rad).

### Minimum Inhibitory Concentration

Fresh bacteria grown overnight on Muller-Hinton Agar plate were resuspended in cation-adjusted Muller-Hinton broth II (MHBII) (Fluka Biochemika, Sigma-Aldrich, France) to an optical density (600 nm) of 0.1 (corresponding to 1 × 10^8^ CFU/mL). This bacterial suspension was adjusted to 1 × 10^6^ CFU/mL in MHBII. Twofolds geometric dilution of each tested antibiotic were performed in 96-well plates using a pipetting robot (ASSIST Plus, INTEGRA Biosciences AG, Zizers, Switzerland). Bacterial inoculates were added to obtain a final concentration of 5 × 10^5^ CFU/mL. Plates were then incubated 16–20 hours at 35°C +/- 1°C in an ambient air incubator. MIC was determined as the lowest drug concentration inhibiting visible bacterial growth. Results were confirmed by an absorbance measurement at 600 nm with an automatic plate-reader (Infinite 200 Pro, Tecan Trading AG, Männedorf, Switzerland). MICs were performed in triplicate.

### Sequential time-kill curve experiment

Bacterial inoculum was prepared by suspension of the bacteria from a 2 hours logarithmic-growth-phase culture in cation-adjusted MHBII, adjusted to a final concentration of 1 × 10^6^ CFU/mL. The experiments were performed in 24-well plates (Greiner Bio-One, Courtaboeuf, France), with an Assist Plus pipetting robot (Integra Biosciences, Zizers, Switzerland). Antibiotics were added to obtain concentrations corresponding to 0.5 × to 16 × MIC for C/T and 0.25 × to 8 × MIC for IMI. To compensate for IMI degradation, (16) doses were added at 9 and 24 hours post inoculation (44% and 35% of the initial dose at 9 and 24 hours, respectively). The 24-well plates were incubated at 35°C for 30 hours under agitation (130 rpm). Bacteria were counted at 0, 3, 6, 24, and 30 hours by agar plating using an automatic plater (easySpiral pro, Interscience, Saint Nom la Bretèche, France). At the start of the experiment, the wells contained 2.2 mL of bacterial suspension, and no less than 1.88 mL after the last sampling at 30 hours. As previously described (18), at 24 hours, a second TKC was started, using bacteria from wells where regrowth was observed in presence of antibiotics. Protocol was identical as the first TKC, except for antibiotics concentration that were increased (up to 64× MIC). The number of CFU was counted after incubation at 35°C for 16 to 20 hours on MH agar plate. Semi-automatic plate reader was used for bacterial count on agar plates (Scan300, Interscience, Saint Nom la Bretèche, France). The limit of quantification was fixed at 200 CFU/mL. At least one growth control, performed without addition of antibiotic, was included in each experiment. All experiments were performed at least three times.

### Antibiotic stability assays

Antibiotic assays (HPLC-MSMS) were performed during TKC experiments to ensure stability of C/T and IMI in MHBII. Procedures are detailed in the Text S2.

### Pharmacodynamic modeling

**Fig 5:**
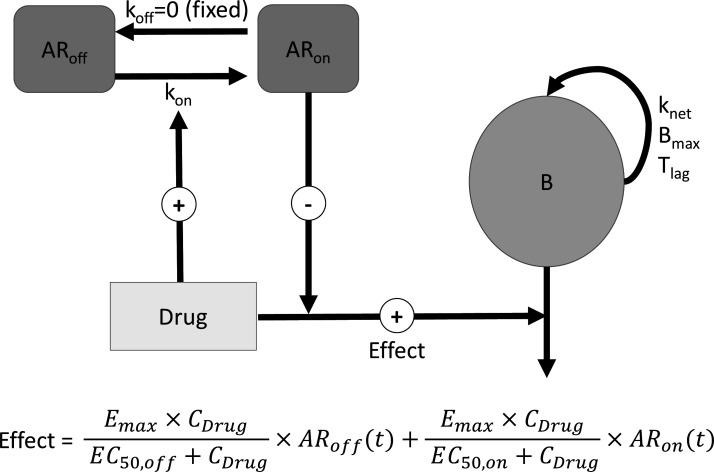
Schematic representation of the final PKPD model. B: Bacterial; AR_on_ and AR_off_: virtual compartments representing the fraction of adapted and non-adapted bacteria, respectively; k_off_: rate constant for reversal of adaptive resistance; k_on_: rate constant for development of adaptive resistance; T_lag_: growth lag time; B_max_: maximal bacterial density in the system; k_net_: difference between growth and death of bacteria in the absence of antibiotic; E_max_: maximum rate constant for drug effect; EC_50,off_: drug concentration necessary to reach 50% of E_max_ when 0% of bacteria are adapted; EC_50,on_: drug concentration necessary to reach 50% of E_max_ when 100% of bacteria are adapted. Parameters are defined in Tables S4 to S6 and model equations.

Data were analyzed using a PKPD model with adaptation (Fig. 5). Drug (C/T or IMI) effect follows Equation 1:


[1] $$Effect=\;\frac{E_{max}\times C_{Drug}}{\;EC_{50,\;\;off}+C_{Drug}}\times AR_{off}+\frac{E_{max}\times C_{Drug}}{\;EC_{50,\;\;on}+C_{Drug}}\times AR_{on}$$

where E_max_ corresponds to the maximal effect rate constant, C_Drug_ the drug (C/T or IMI) concentration, EC_50,off_ the drug concentration necessary to reach 50% of E_max_ in non-adapted bacteria, AR_off_ the proportion of non-adapted bacteria, EC_50,on_ the drug concentration necessary to reach 50% of E_max_ in adapted bacteria and AR_on_ the proportion of adapted bacteria.

For each strain, the bactericidal effect of both drugs can be described by an E_max_ model for both the adapted and non-adapted bacteria, with a common E_max_ but distinct EC_50_ values (respectively, EC_50,off_ at time zero, and EC_50,on_ at time infinity)

Comparisons of EC_50,off_ values of the various mutants with that of the reference PAO1 characterize the acquired resistance, whereas comparisons between EC_50,off_ and EC_50,on_ for a particular strain reflects adaptive resistance. The fractions of non-adapted AR_off_ and adapted AR_on_ bacteria as a function of time were computed using the equations 2 and 3,


[2] $$AR_{off}=e^{-k_{on}\times t}$$

[3] $$AR_{on}=1-AR_{off}$$

where k_on_ represents the adaptation rate. To study the change in EC_50_ value from EC_50,off_ before adaptation starts, to EC_50,on_ when 100% of bacteria are adapted the following procedure was followed: the drug effect at any given time t (effect) was computed for a wide range of concentrations [i.e., C_Drug_∈ (0,EC_50,on_)] according to equation (1). The concentration for which Effect = 50% E_max_ at t was found and called EC_50,off→on_. The procedure was reproduced for all times between the beginning and the end of TKCs [i.e., t ∈(0,54)] to yield EC_50,off→on_ at all time-points. Details and full differential equations are provided in Text S1.

## Data Availability

The whole-genome sequences of the strains analyzed in this study are available under BioProject accession number PRJNA998736 (strains PaS and PaR).

## SUPPLEMENTAL MATERIAL

Supplemental data aac.00480-23-s0001.docx contains supplement table S1 to S6, supplemental Figure S1 to s3 and supplemental text S1 and S2.

ASM does not own the copyrights to Supplemental Material that may be linked to, or accessed through, an article. The authors have granted ASM a non-exclusive, world-wide license to publish the Supplemental Material files. Please contact the corresponding author directly for reuse.
