# Supplementary material for: Characterization of Pseudomonas aeruginosa resistance to ceftolozane-tazobactam due to ampC and/or ampD mutations observed during treatment using semi-mechanistic PKPD modeling
Source: Antimicrob Agents Chemother. 2023 Sep 11;67(10):e00480-23. doi: 10.1128/aac.00480-23 (PMC10583683; doi:10.1128/aac.00480-23)
Supplement: Supplemental data — contains supplement table S1 to S6, supplemental Figure S1 to s3 and supplemental text S1 and S2. [file aac.00480-23-s0001.docx]

**Table S1.** Mutations found in PaR compared to PaS.

| **PAO1 locus - Gene name (if available)** | **Nucleic acid position on PAO1 reference genome** | **Change** | **Polymorphism Type** | **Product** | **Mutation type** | **Amino-acid change** |
| --- | --- | --- | --- | --- | --- | --- |
| PA0040 | 41636 | +G | Insertion | conserved hypothetical protein | frameshift |  |
|  | 42093 | T -> C | Substitution |  | silent mutation |  |
| PA0041 | 44133 | T -> C | Substitution | probable hemagglutinin | substitution | V407A |
|  | 45794 | A -> G | Substitution |  | substitution | I961V |
| PA0074 - ppkA | 89181 | C -> T | Substitution | serine/threonine protein kinase PpkA | silent mutation |  |
|  | 89216 | G -> T | Substitution |  | substitution | L295M |
| PA0095 - vgrG1b | 116738 | C -> A | Substitution | Type VI secretion system | silent mutation |  |
| PA0216 - madM | 244458 | C -> T | Substitution | malonate transporter MadM | silent mutation |  |
| PA0259 - tla3 | 290464 | T -> A | Substitution | Type 6 lipase adaptor, Tla3 | substitution | I181F |
|  | 290812 | T -> C | Substitution |  | substitution | T65A |
| PA0317 | 357485 | -G | Deletion | D-2-hydroxyglutarate dehydrogenase | frameshift |  |
| PA0319 | 359913 | +CCGATTAAGCTTCGCTTAAGCG | Insertion | hypothetical protein | sequence duplication |  |
| PA0435 | 488326 | -C | Deletion | hypothetical protein | frameshift |  |
| PA0450 | 506965 | A -> G | Substitution | probable phosphate transporter | substitution | V96A |
| PA0458 - yieO | 517132 | C -> T | Substitution | probable major facilitator superfamily (MFS) transporter | substitution | V111I |
| PA0459 - clpC | 519729 | C -> G | Substitution | probable ClpA/B protease ATP binding subunit | silent mutation |  |
| PA0508 | 569364 | C -> G | Substitution | probable acyl-CoA dehydrogenase | silent mutation |  |
|  | 570066 | G -> A | Substitution |  | silent mutation |  |
|  | 570108 | G -> C | Substitution |  | silent mutation |  |
|  | 570174 | G -> T | Substitution |  | silent mutation |  |
|  | 570178 | C -> T | Substitution |  | silent mutation |  |
|  | 570186 | C -> G | Substitution |  | silent mutation |  |
|  | 570201 | A -> C | Substitution |  | silent mutation |  |
|  | 570213 | C -> T | Substitution |  | silent mutation |  |
|  | 570218 | G -> C | Substitution |  | substitution | G406A |
|  | 570233 | T -> C | Substitution |  | substitution | I411T |
| PA0575 - rmcA | 631319 | C -> T | Substitution | redox regulator of c_di_GMP, RmcA | silent mutation |  |
| PA0576 - rpoD | 634781 | -G | Deletion | sigma factor RpoD | frameshift |  |
| PA0603 - agtA | 664890 | G -> A | Substitution | AgtA | silent mutation |  |
|  | 664914 | C -> G | Substitution |  | silent mutation |  |
|  | 664917 | C -> G | Substitution |  | silent mutation |  |
|  | 664953 | G -> C | Substitution |  | silent mutation |  |
|  | 664975 | G -> A | Substitution |  | silent mutation |  |
|  | 664986 | C -> T | Substitution |  | silent mutation |  |
| PA0604 - agtB | 666325 | T -> C | Substitution | AgtB | silent mutation |  |
| PA0606 - agtD | 668671 | T -> C | Substitution | AgtD | silent mutation |  |
|  | 668715 | G -> C | Substitution |  | silent mutation |  |
|  | 668730 | T -> C | Substitution |  | silent mutation |  |
|  | 669171 | G -> C | Substitution |  | silent mutation |  |
| PA0616 | 676579 | C -> T | Substitution | hypothetical protein | silent mutation |  |
|  | 676603 | C -> T | Substitution |  | silent mutation |  |
| PA0640 | 694825 | G -> C | Substitution | probable bacteriophage protein | silent mutation |  |
| PA0641 | 697301 | G -> A | Substitution | probable bacteriophage protein | silent mutation |  |
| PA0667 - mepM | 719652 | C -> T | Substitution | MepM (Peptidoglycan cross-link hydrolase) | substitution | A141T |
| PA0685 - hxcQ | 744313 | T -> C | Substitution | HxcQ | silent mutation |  |
| PA0690 - pdtA | 750118 | T -> G | Substitution | phosphate depletion regulated TPS partner A, PdtA | silent mutation |  |
| PA0724 - coaA | 792326 | C -> T | Substitution | probable coat protein A of bacteriophage Pf1 | silent mutation |  |
|  | 792344 | G -> A | Substitution |  | silent mutation |  |
|  | 792408 | C -> A | Substitution |  | substitution | Q347K |
|  | 792443 | T -> C | Substitution |  | silent mutation |  |
|  | 792455 | C -> T | Substitution |  | silent mutation |  |
| PA0728 - intF4 | 795798 | G -> C | Substitution | probable bacteriophage integrase | silent mutation |  |
| PA0730 | 797950 | A -> G | Substitution | probable transferase | substitution | G293A |
| PA0767 - lepA | 836331 | -G | Deletion | GTP-binding protein LepA | frameshift |  |
| PA0796 - prpB | 874325 | -C | Deletion | carboxyphosphonoenolpyruvate phosphonomutase | frameshift |  |
| PA0847 | 926923 | A -> G | Substitution | diguanylate cyclase | silent mutation |  |
| PA0933 - ygcA | 1022023 | T -> C | Substitution | probable RNA methyltransferase | silent mutation |  |
| PA0960 | 1046620 | G -> A | Substitution | hypothetical protein | silent mutation |  |
| PA0987 | 1068573 | G -> A | Substitution | conserved hypothetical protein | substitution | A29T |
| PA1094 - fliD | 1185899 | G -> C | Substitution | flagellar capping protein FliD | silent mutation |  |
| PA1101 - fliF | 1192682 | A -> G | Substitution | Flagella M-ring outer membrane protein precursor | substitution | Y93C |
|  | 1193474 | G -> A | Substitution |  | substitution | G357D |
| PA1194 | 1294869 | A -> G | Substitution | probable amino acid permease | substitution | V454G |
| PA1196 - ddaR | 1297573 | C -> T | Substitution | transcriptional regulator DdaR | substitution | P89S |
| PA1214 | 1315301 | C -> T | Substitution | hypothetical protein | substitution | A206T |
| PA1237 | 1338469 | T -> C | Substitution | probable multidrug resistance efflux pump | substitution | K205R |
| PA1322 - pfuA | 1433761 | C -> T | Substitution | probable TonB-dependent receptor | silent mutation |  |
| PA1360 | 1472208 | G -> A | Substitution | conserved hypothetical protein | substitution | A180T |
| PA1378 | 1495821 | C -> T | Substitution | hypothetical protein | substitution | R63W |
| PA1382 - xqhB | 1500576 | C -> T | Substitution | probable type II secretion system protein | silent mutation |  |
|  | 1500585 | T -> C | Substitution |  | silent mutation |  |
|  | 1500655 | C -> T | Substitution |  | silent mutation |  |
| PA1450 | 1578686 | T -> G | Substitution | conserved hypothetical protein | silent mutation |  |
| PA1477 - ccmC | 1604115 | CA -> AG | Substitution | heme exporter protein CcmC | substitution | Q149R |
| PA1480 - ccmF | 1605129 | T -> C | Substitution | cytochrome C-type biogenesis protein CcmF | substitution | R199N |
|  | 1605682 | CG -> AA | Substitution |  | substitution | R199N |
| PA1483 - cycH | 1608118 | A -> T | Substitution | cytochrome c-type biogenesis protein | silent mutation |  |
| PA1497 | 1624814 | -G | Deletion | probable transporter | frameshift |  |
| PA1511 - vgrG2a | 1641926 | C -> T | Substitution | VgrG2a | silent mutation |  |
|  | 1642652 | T -> C | Substitution |  | silent mutation |  |
| PA1586 - sucB | 1727771 | C -> T | Substitution | dihydrolipoamide succinyltransferase (E2 subunit) | silent mutation |  |
| PA1595 | 1737164 | +GCG | Insertion | hypothetical protein | substitution | +A insertion |
| PA1601 | 1744135 | -G | Deletion | probable aldehyde dehydrogenase | frameshift |  |
| PA1609 - fabB | 1751985 | -C | Deletion | beta-ketoacyl-ACP synthase I | frameshift |  |
| PA1631 | 1775397 | T -> C | Substitution | probable acyl-CoA dehydrogenase | silent mutation |  |
|  | 1775443 | T -> C | Substitution | - | silent mutation |  |
|  | 1775445 | G -> C | Substitution | - | silent mutation |  |
|  | 1775446 | A -> G | Substitution | - | substitution | I282T |
|  | 1775448 | C -> T | Substitution | - | silent mutation |  |
|  | 1775454 | G -> C | Substitution | - | silent mutation |  |
|  | 1775460 | T -> G | Substitution | - | silent mutation |  |
|  | 1775472 | G -> A | Substitution | - | silent mutation |  |
| PA1648 | 1795755 | A -> G | Substitution | probable oxidoreductase | substitution | Y45H |
| PA1719 - pscF | 1863010 | C -> T | Substitution | type III export protein PscF | substitution | Q83-Stop |
| PA1725 - pscL | 1866071 | C -> T | Substitution | type III export protein PscL | substitution | A193V |
| PA1742 - pauD2 | 1886758 | -G | Deletion | Glutamine amidotransferase class I | frameshift |  |
| PA1760 | 1903772 | A -> G | Substitution | probable transcriptional regulator | substitution | A750G |
| PA1807 - nppD | 1962550 | -G | Deletion | NppD | frameshift |  |
| PA1812 - mltD | 1970011 | C -> T | Substitution | membrane-bound lytic murein transglycosylase D precursor | substitution | R410Q |
| PA1874 | 2039968 | C -> T | Substitution | hypothetical protein | silent mutation |  |
|  | 2039977 | G -> A | Substitution |  | silent mutation |  |
|  | 2039983 | -T | Deletion |  | frameshift |  |
| PA1908 | 2079758 | -C | Deletion | probable major facilitator superfamily (MFS) transporter | frameshift |  |
| PA1941 | 2124335 | G -> A | Substitution | hypothetical protein | silent mutation |  |
| PA1984 - exaC | 2170866 | T -> C | Substitution | NAD+ dependent aldehyde dehydrogenase ExaC | silent mutation |  |
| PA1993 - yhhS | 2180615 | -C | Deletion | probable major facilitator superfamily (MFS) transporter | frameshift | premature stop codon |
| PA2001 - atoB | 2187373 | C -> T | Substitution | acetyl-CoA acetyltransferase | silent mutation |  |
|  | 2187427 | G -> A | Substitution |  | silent mutation |  |
|  | 2187724 | G -> A | Substitution |  | silent mutation |  |
|  | 2187733 | C -> T | Substitution |  | silent mutation |  |
|  | 2187739 | C -> A | Substitution |  | silent mutation |  |
|  | 2187741 | T -> C | Substitution |  | substitution | V226A |
|  | 2187748 | C -> G | Substitution |  | silent mutation |  |
| PA2060 - sppC | 2257047 | -C | Deletion | ABC transporter permease, SppC | frameshift | premature stop codon |
| PA2074 | 2279538 | C -> G | Substitution | hypothetical protein | substitution | A186G |
| PA2089 | 2300481 | -C | Deletion | hypothetical protein | frameshift | premature stop codon |
| PA2094 | 2305245 | T -> C | Substitution | probable transmembrane sensor | substitution | V141A |
| PA2097 | 2309425 | C -> T | Substitution | probable flavin-binding monooxygenase | substitution | A490V |
| PA2142 - yhxC | 2357145 | T -> C | Substitution | probable short-chain dehydrogenase | substitution | W145R |
| PA2176 | 2396909 | C -> T | Substitution | hypothetical protein | substitution | R193H |
| PA2187 | 2407338 | C -> T | Substitution | hypothetical protein | substitution | P35S |
|  | 2407567 | Ac -> GG | Substitution |  | substitution | H111R |
| PA2216 | 2437100 | -T | Deletion | 2-keto-3-deoxy-D-arabinonate dehydratase | frameshift | premature stop codon |
| PA2254 - pvcA | 2482853 | -C | Deletion | paerucumarin biosynthesis protein PvcA | frameshift | premature stop codon |
| PA2291 - oprB2 | 2521516 | A -> G | Substitution | probable glucose-sensitive porin | silent mutation |  |
| PA2302 - ambE | 2533443 | +C | Insertion | AmbE | frameshift | No stop codon |
| PA2329 | 2571029 | G -> A | Substitution | transcriptional regulator MtlR | silent mutation |  |
| PA2337 - mtlR | 2581876 | C -> T | Substitution | probable transcriptional regulator | silent mutation |  |
| PA2359 - sfnR2 | 2606064 | T -> C | Substitution | hypothetical protein | substitution | H320R |
| PA2360 - hsiA3 | 2608133 | C -> T | Substitution | hypothetical protein | substitution | D34N |
| PA2399 - pvdD | 2661979 | G -> A | Substitution | pyoverdine synthetase D | silent mutation |  |
| PA2402 - pvdI | 2677600 | G -> A | Substitution | pyoverdine peptide synthetase | silent mutation |  |
|  | 2677702 | G -> A | Substitution |  | silent mutation |  |
|  | 2683794 | -G | Deletion |  | frameshift | premature stop codon |
|  | 2684436 | G -> A | Substitution |  | silent mutation |  |
|  | 2684675 | T -> C | Substitution |  | substitution | Y835C |
|  | 2685109 | G -> C | Substitution |  | silent mutation |  |
| PA2457 | 2757616 | G -> T | Substitution | hypothetical protein | silent mutation |  |
| PA2460 | 2760358 | T -> G | Substitution | hypothetical protein | substitution | T88P |
|  | 2760422 | T -> G | Substitution |  | substitution | E66D |
| PA2462 | 2770085 | T -> G | Substitution | hypothetical protein | substitution | D2907A |
|  | 2775580 | -C | Deletion |  | frameshift | premature stop codon |
| PA2509 - catB | 2826023 | -G | Deletion | muconate cycloisomerase I | frameshift | No stop codon |
| PA2515 - xylL | 2832395 | G -> A | Substitution | cis-1,2-dihydroxycyclohexa-3,4-diene carboxylate dehydrogenase | substitution | P246S |
| PA2539 - ynbD | 2866879 | A -> G | Substitution | conserved hypothetical protein | substitution | C210R |
| PA2557 | 2890574 | T -> C | Substitution | probable AMP-binding enzyme | substitution | I493V |
|  | 2890587 | G -> C | Substitution |  | silent mutation |  |
|  | 2890589 | G -> T | Substitution |  | silent mutation |  |
|  | 2890633 | T -> G | Substitution |  | substitution | H473T |
|  | 2890635 | A -> G | Substitution |  | silent mutation |  |
|  | 2890637 | T -> A | Substitution |  | substitution | T472S |
|  | 2890650 | C -> T | Substitution |  | silent mutation |  |
|  | 2890655 | G -> A | Substitution |  | silent mutation |  |
|  | 2890805 | C -> T | Substitution |  | substitution | D416N |
|  | 2891757 | C -> G | Substitution |  | silent mutation |  |
|  | 2891763 | G -> T | Substitution |  | silent mutation |  |
|  | 2891769 | A -> C | Substitution |  | silent mutation |  |
| PA2600 | 2943679 | -C | Deletion | hypothetical protein | frameshift | premature stop codon |
| PA2681 | 3028523 | T -> C | Substitution | probable transcriptional regulator | substitution | V150A |
| PA2782 - bamI | 3139168 | C -> T | Substitution | biofilm-associated metzincin Inhibitor, BamI | substitution | A53V |
| PA2951 - etfA | 3310870 | A -> C | Substitution | electron transfer flavoprotein alpha-subunit | silent mutation |  |
|  | 3310993 | G -> A | Substitution |  | silent mutation |  |
|  | 3311056 | G -> A | Substitution |  | silent mutation |  |
|  | 3311080 | G -> A | Substitution |  | silent mutation |  |
|  | 3311101 | G -> A | Substitution |  | silent mutation |  |
|  | 3311113 | G -> A | Substitution |  | silent mutation |  |
|  | 3311128 | G -> C | Substitution |  | silent mutation |  |
|  | 3311413 | G -> C | Substitution |  | silent mutation |  |
|  | 3311455 | G -> A | Substitution |  | silent mutation |  |
|  | 3311461 | C -> T | Substitution |  | silent mutation |  |
|  | 3311475 | T -> C | Substitution |  | substitution | N83D |
|  | 3311491 | G -> C | Substitution |  | silent mutation |  |
|  | 3311493 | T -> C | Substitution |  | substitution | I77V |
| PA2952 | 3311973 | G -> A | Substitution | etfB | silent mutation |  |
|  | 3312060 | G -> A | Substitution |  | silent mutation |  |
|  | 3312066 | G -> C | Substitution |  | silent mutation |  |
|  | 3312069 | C -> G | Substitution |  | silent mutation |  |
|  | 3312076 | G -> C | Substitution |  | substitution | A132G |
|  | 3312078 | G -> C | Substitution |  | silent mutation |  |
|  | 3312152 | T -> G | Substitution |  | substitution | K107Q |
| PA2975 - rluC | 3331544 | T -> C | Substitution | ribosomal large subunit pseudouridine synthase C | substitution | H254R |
| PA3075 | 3449263 | -C | Deletion | hypothetical protein | frameshift | premature stop codon |
| PA3121 - leuC | 3504021 | -C | Deletion | 3-isopropylmalate dehydratase large subunit | frameshift | premature stop codon |
| PA3194 - edd | 3586935 | T -> C | Substitution | phosphogluconate dehydratase | silent mutation |  |
| PA3234 - yjcG | 3622165 | G -> A | Substitution | probable sodium:solute symporter | silent mutation |  |
| PA3269 | 3659059 | -G | Deletion | probable transcriptional regulator | frameshift | premature stop codon |
| PA3290 - tle1 | 3683591 | A -> G | Substitution | Tle1 | silent mutation |  |
| PA3294 - vgrG4a | 3687097 | G -> A | Substitution | VgrG4a | silent mutation |  |
|  | 3687462 | A -> G | Substitution |  | substitution | W397R |
|  | 3688552 | G -> A | Substitution |  | silent mutation |  |
| PA3346 - hsbR | 3757855 | +C | Insertion | HptB-dependent secretion and biofilm regulator HsbR | frameshift | No stop codon |
| PA3360 | 3771904 | G -> A | Substitution | probable secretion protein | substitution | P220S |
| PA3364 - amiC | 3774988 | G -> A | Substitution | aliphatic amidase expression-regulating protein | substitution | Q250-stop |
| PA3465 | 3876560 | T -> C | Substitution | conserved hypothetical protein | substitution | L122P |
| PA3481 | 3894909 | C -> T | Substitution | conserved hypothetical protein | substitution | M95I |
| PA3485 - tsi3 | 3899935 | A -> G | Substitution | Tsi3 | silent mutation |  |
| PA3491 - rnfC | 3909694 | A -> G | Substitution | probable ferredoxin | substitution | Y428C |
| PA3522 - mexQ | 3940376 | T -> C | Substitution | MexQ | substitution | I760V |
| PA3593 | 4027758 | T -> C | Substitution | probable acyl-CoA dehydrogenase | substitution | D461V |
| PA3635 - eno | 4069645 | C -> T | Substitution | enolase | substitution | A108T |
| PA3667 | 4105900 | -C | Deletion | probable pyridoxal-phosphate dependent enzyme | frameshift | premature stop codon |
| PA3689 - yhdM | 4131418 | T -> C | Substitution | probable transcriptional regulator | substitution | K2R |
| PA3690 | 4133431 | C -> G | Substitution | hypothetical protein | silent mutation |  |
|  | 4133449 | C -> T | Substitution |  | silent mutation |  |
|  | 4133450 | G -> T | Substitution |  | substitution | A639S |
|  | 4133455 | G -> C | Substitution |  | silent mutation |  |
|  | 4133458 | C -> G | Substitution |  | silent mutation |  |
| PA3867 | 4330367 | A -> G | Substitution | probable DNA invertase | substitution | D16G |
| PA3869 | 4333029 | C -> A | Substitution | hypothetical protein | substitution | P126Q |
| PA3883 | 4349531 | C -> T | Substitution | probable short-chain dehydrogenase | silent mutation |  |
|  | 4349534 | C -> T | Substitution |  | silent mutation |  |
|  | 4349793 | A -> C | Substitution |  | substitution | M98L |
|  | 4349810 | G -> A | Substitution |  | silent mutation |  |
|  | 4349819 | G -> A | Substitution |  | silent mutation |  |
| PA3952 | 4432934 | A -> G | Substitution | hypothetical protein | silent mutation |  |
| PA3958 | 4438344 | G -> A | Substitution | hypothetical protein | substitution | V319I |
| PA3974 - ladS | 4453781 | C -> T | Substitution | Lost Adherence Sensor, LadS | silent mutation |  |
| PA3992 - sltB3 | 4472810 | C -> T | Substitution | SltB3 (soluble lytic (exolytic) transglycosylase) | substitution | A227V |
| PA3994 | 4475784 | -C | Deletion | probable epoxide hydrolase | frameshift | premature stop codon |
| PA4097 - ydjL | 4580049 | T -> C | Substitution | probable alcohol dehydrogenase (Zn-dependent) | silent mutation |  |
|  | 4580061 | T -> C | Substitution |  | silent mutation |  |
|  | 4580211 | A -> G | Substitution |  | silent mutation |  |
|  | 4583948 | T -> C | Substitution |  | substitution | L366P |
| PA4110 - ampC | 4594576 | G -> A | Substitution | beta-lactamase precursor | substitution | G183D |
| PA4112 | 4596999 | -G | Deletion | probable sensor/response regulator hybrid | frameshift | premature stop codon |
| PA4152 - acoC | 4646672 | A -> G | Substitution | probable hydrolase | substitution | N360S |
| PA4165 | 4661737 | C -> A | Substitution | probable transcriptional regulator | substitution | G106V |
| PA4172 | 4668850 | T -> C | Substitution | probable nuclease | substitution | F121L |
| PA4186 | 4683199 | T -> C | Substitution | hypothetical protein | substitution | Y320H |
| PA4212 - phzC1 | 4714981 | G -> C | Substitution | phenazine biosynthesis protein PhzC | silent mutation |  |
| PA4223 - pchH | 4729436 | C -> T | Substitution | probable ATP-binding component of ABC transporter | silent mutation |  |
| PA4318 | 4846888 | T -> C | Substitution | hypothetical protein | substitution | V59A |
| PA4371 | 4900619 | -C | Deletion | hypothetical protein | frameshift | premature stop codon |
| PA4522 - ampD | 5064872 | G -> A | Substitution | beta-lactamase expression regulator AmpD | substitution | H157Y |
| PA4621 | 5180743 | T -> C | Substitution | probable oxidoreductase | substitution | K509R |
| PA4625 - cdrA | 5188259 | A -> C | Substitution | cyclic diguanylate-regulated TPS partner A, CdrA | silent mutation |  |
|  | 5188454 | T -> C | Substitution |  | silent mutation |  |
|  | 5189063 | G -> A | Substitution |  | silent mutation |  |
| PA4750 - folP | 5335494 | G -> A | Substitution | dihydropteroate synthase | silent mutation |  |
| PA4793 | 5380854 | A -> G | Substitution | hypothetical protein | substitution | Y92C |
| PA4816 | 5407315 | -G | Deletion | hypothetical protein | frameshift | No stop codon |
| PA4843 - gcbA | 5436187 | -C | Deletion | GcbA | frameshift | premature stop codon |
| PA4902 | 5500842 | G -> A | Substitution | probable transcriptional regulator | substitution | W139-Stop |
| PA4999 - waaL | 5617338 | A -> G | Substitution | O-antigen ligase, WaaL | silent mutation |  |
|  | 5617341 | C -> G | Substitution |  | silent mutation |  |
|  | 5617344 | G -> T | Substitution |  | silent mutation |  |
| PA5037 | 5672738 | A -> C | Substitution | hypothetical protein | silent mutation |  |
| PA5060 - phaF | 5700937 | -T | Deletion | polyhydroxyalkanoate synthesis protein PhaF | frameshift | No stop codon |
| PA5072 - mcpK | 5710658 | C -> G | Substitution | McpK | silent mutation |  |
| PA5253 - algP | 5915270 | A -> G | Substitution | alginate regulatory protein AlgP | substitution | S278P |
| PA5275 - cyaY | 5940781 | +C | Insertion | conserved hypothetical protein | frameshift | No stop codon |
| PA5346 - sadB | 6015187 | +C | Insertion | SadB | frameshift | No stop codon |
| PA5496 - nrdJb | 6188430 | G -> C | Substitution | class II (cobalamin-dependent) ribonucleotide-diphosphate reductase subunit, NrdJb | silent mutation |  |
|  | 6188433 | A -> C | Substitution | - | silent mutation |  |
|  | 6188444 | T -> C | Substitution | - | substitution | I138V |
|  | 6188451 | T -> C | Substitution | - | silent mutation |  |
|  | 6188469 | G -> A | Substitution | - | silent mutation |  |
|  | 6188490 | G -> C | Substitution | - | silent mutation |  |
|  | 6188499 | G -> C | Substitution | - | silent mutation |  |
|  | 6188502 | A -> C | Substitution | - | silent mutation |  |
|  | 6188532 | A -> C | Substitution | - | silent mutation |  |
|  | 6188547 | G -> C | Substitution | - | silent mutation |  |
|  | 6188553 | A -> C | Substitution | - | silent mutation |  |
|  | 6188556 | T -> C | Substitution | - | silent mutation |  |
|  | 6188574 | A -> G | Substitution | - | silent mutation |  |
| PA5525 | 6218316 | G -> A | Substitution | probable transcriptional regulator | substitution | A73T |
| PA5528 | 6220232 | G -> A | Substitution | hypothetical protein | substitution | R170C |
| PA5531 - tonB1 | 6225275 | C -> G | Substitution | TonB1 | silent mutation |  |
| PA5555 - atpG | 6250426 | -C | Deletion | ATP synthase gamma chain | frameshift | premature stop codon |

**Table S2.** PAO1 and mutant-derived strains parameter estimates for ceftolozane/tazobactam with 95% confidence intervals determined by SIR for the final model.

| **PAO1 and mutant-derived strains PKPD parameters** | | | | | |
| --- | --- | --- | --- | --- | --- |
| **Parameter** | **Description** | **PAO1** | **PAO1-AmpC^G183D^** | **PAO1-AmpD^H157Y^** | **PAO1-AmpC^G183D^/AmpD^H157Y^** |
| k_net_ (h^-1^) | Difference between growth and death of bacteria in the absence of antibiotic | 1.38 [1.2 - 1.58] | 0.823 [0.762 - 0.877] | 0.873 [0.806 - 0.933] | 0.824 [0.799 - 0.852] |
| B_max_ log_10_(CFU/mL) | Maximal bacterial density in the system | 10 [9.9 - 10.1] | 9.97 [9.87 - 10.1] | 9.98 [9.91 - 10.1] | 10 [9.84 - 10.2] |
| T_lag1_ (h) | Growth lag time during the first TKC | 3.24 [3.12 - 3.42] | 0 (fixed) | 0 (fixed) | 0 (fixed) |
| T_lag2_ (h) | Growth lag time during the second TKC | 3.18 [3.09 - 3.27] | 3.09 [2.92 - 3.31] | 2.8 [2.68 - 2.91] | 2.94 [2.85 - 3.02] |
| E_max_ (h^-1^) | Maximum rate constant for drug effect | 1.94 [1.82 - 2.07] | | | |
| EC_50,off_ (mg/L) | Drug concentration necessary to reach 50% of E_max_ when 0% of bacteria are adapted | 0.0444 [0.00818 - 0.104] | 0.0606 [0.0294 - 0.0988] | 0.181 [0.121 - 0.263] | 1.29 [0.499 - 2.55] |
| EC_50,on_ (mg/L) | Drug concentration necessary to reach 50% of E_max_ when 100 % of bacteria are adapted | 0.551 [0.408 - 0.743] | 19.4 [15.8 - 23.4] | 2.2 [1.83 - 2.59] | 70.8 [60.5 - 83.1] |
| k_on_ (h^-1^*mg^-1^*L) | Rate constant for development of adaptive resistance | 0.0897 [0.0782 - 0.103] | | | |
| k_off_ (h^-1^) | Rate constant for reversal of adaptive resistance | 0 (fixed) | | | |
| σ^2^ | Variance of the residual unexplained variability | 2.76 [2.15 - 3.61] | 1.2 [0.984 - 1.48] | 1.07 [0.942 - 1.23] | 0.914 [0.749 - 1.12] |

**Table S3.** Clinical strains and PaR mutant-derived parameter estimates for ceftolozane/tazobactam with 95% confidence intervals determined by SIR for the final model.

| **Clinical isolates and PaR mutant-derived strains PKPD parameters** | | | | |
| --- | --- | --- | --- | --- |
| **Parameter** | **Description** | **PaS** | **PaR-AmpC^PaS^/AmpD^PaS^** | **PaR** |
| k_net_ (h^-1^) | Difference between growth and death of bacteria in the absence of antibiotic | 0.891 [0.817 - 0.969] | 0.768 [0.617 - 0.893] | 0.901 [0.838 - 0.964] |
| B_max_ log_10_(CFU/mL) | Maximal bacterial density in the system | 9.44 [9.35 - 9.54] | 9.07 [9.02 - 9.12] | 9.38 [9.23 - 9.53] |
| T_lag1_ (h) | Growth lag time during the first TKC | 0 (fixed) | 0 (fixed) | 0 (fixed) |
| T_lag2_ (h) | Growth lag time during the second TKC | 0 (fixed) | 0 (fixed) | 2.82 [2.61 - 2.98] |
| E_max_ (h^-1^) | Maximum rate constant for drug effect | 3.09 [2.91 - 3.29] | | |
| EC_50,off_ (mg/L) | Drug concentration necessary to reach 50% of E_max_ when 0% of bacteria are adapted | 1.36 [1.04 - 1.73] | 6.77 [5.37 - 8.17] | 80.5 [66.4 - 92] |
| EC_50,on_ (mg/L) | Drug concentration necessary to reach 50% of E_max_ when 100 % of bacteria are adapted | 155 [124 - 192] | 269 [220 - 345] | 420 [371 - 485] |
| k_on_ (h^-1^*mg^-1^*L) | Rate constant for development of adaptive resistance | 0.0614 [0.054 - 0.0706] | | |
| k_off_ (h^-1^) | Rate constant for reversal of adaptive resistance | 0 (fixed) | | |
| σ^2^ | Variance of the residual unexplained variability | 1.06 [0.923 - 1.22] | 0.963 [0.863 - 1.07] | 0.788 [0.662 - 0.987] |

**Table S4.** PAO1 and mutant-derived strains parameter estimates for imipenem with 95% confidence intervals determined by SIR for the final model.

| **PAO1 and mutant-derived strains PKPD parameters** | | | | | |
| --- | --- | --- | --- | --- | --- |
| **Parameter** | **Description** | **PAO1** | **PAO1-AmpC^G183D^** | **PAO1-AmpD^H157Y^** | **PAO1-AmpC^G183D^/AmpD^H157Y^** |
| k_net_ (h^-1^) | Difference between growth and death of bacteria in the absence of antibiotic | 2.12 [1.79 – 2.48] | 0.684 [0.592 - 0.785] | 1.87 [1.61 – 2.22] | 1.11 [1.02 – 1.19] |
| B_max_ log_10_(CFU/mL) | Maximal bacterial density in the system | 9.93 [9.74 - 10.1] | 9.95 [9.89 - 10.0] | 9.63 [9.41 – 9.83] | 9.86 [9.78 – 9.94] |
| T_lag1_ (h) | Growth lag time during the first TKC | 0 (fixed) | 2.92 [2.57 - 3.21] | 5.6 [5.14 - 6.04] | 2.73 [2.5 - 2.91] |
| T_lag2_ (h) | Growth lag time during the second TKC | 5.81 [5.4 - 6.15] | 0 (fixed) | 3.32 [3.23 - 3.42] | 4.84 [3.56 - 5.83] |
| E_max_ (h^-1^) | Maximum rate constant for drug effect | 4.71 [4.37 – 5.08] | | | |
| EC_50,off_ (mg/L) | Drug concentration necessary to reach 50% of E_max_ when 0% of bacteria are adapted | 0.411 [0.250 - 0.638] | 1.86 [1.49 – 2.26] | 2.03 [1.58 – 2.64] | 0.494 [0.397 – 0.603] |
| EC_50,on_ (mg/L) | Drug concentration necessary to reach 50% of E_max_ when 100 % of bacteria are adapted | 24.8 [21.3 - 29.3] | 2.25 [1.91 - 2.57] | 7.68 [4.62 - 11.1] | 3.29 [2.84 - 3.80] |
| k_on_ (h^-1^*mg^-1^*L) | Rate constant for development of adaptive resistance | 0.0640 [0.0496 – 0.0856] | | | |
| k_off_ (h^-1^) | Rate constant for reversal of adaptive resistance | 0 (fixed) | | | |
| σ^2^ | Variance of the residual unexplained variability | 1.21 [0.972 - 1.46] | 1.2 [1.1 - 1.31] | 2.52 [1.88 - 3.53] | 0.351 [0.301 - 0.405] |

**Table S5. Strains and plasmid used in this study**. KAN: kanamycin, GEN: gentamicin, C/T: ceftolozane/tazobactam, MDR: multidrug resistant.

| Strain | Description | Resistance | Reference (PMID) |
| --- | --- | --- | --- |
| *E. coli* JKE201 | Cloning strain | KAN | 29073136 |
| *E. coli* SM10 | Cloning strain | KAN |  |
| *P. aeruginosa* PAO1 | ATCC 15692 | none |  |
| PAO1-AmpC^G183D^ | PAO1 *ampC* G548A substitution | none | This study |
| PAO1-AmpD^H157Y^ | PAO1 *ampD* C469T substitution | none | This study |
| PAO1-AmpC^G183D^/AmpD^H157Y^ | PAO1 *ampC* G548A + *ampD* C469T substitution | none | This study |
| PaS | Clinical isolate before the C/T treatment | MDR | This study |
| PaR | Clinical isolate after two weeks of C/T treatment | MDR + C/T | This study |
| PaR-AmpC^PaS^ | PaR with *ampC* cloned from PaS | none | This study |
| PaR-AmpD^PaS^ | PaR with *ampD* cloned from PaS | none | This study |
| PaR-AmpC^PaS^/AmpD^PAS^ | PaR with *ampC* and *ampD* cloned from PaS | none | This study |

| Plasmid | Description | Resistance | Reference (PMID) |
| --- | --- | --- | --- |
| pEXG2 | suicide vector for gene deletion in P.  aeruginosa PAO1 | GEN | 15911752 |
| pFOG | suicide vector for gene edition in clinical strain PaR | GEN | 32448155 |
| pEXG2 AmpC^G183D^ | suicide vector for G548A substitution in *ampC (*PAO1 background) | GEN | This study |
| pEXG2 AmpD^H157Y^ | suicide vector for C469T substitution in *ampD* (PAO1 background) | GEN | This study |
| pFOG ampC^PaS^ | suicide vector for cloning of *ampC* from PaS in PaR background) | GEN | This study |
| pFOG ampD^PaS^ | suicide vector for cloning of *ampD* from PaS in PaR background) | GEN | This study |

**Table S6. Primers used in this study.**

| Name | Description | Template | Sequence (5’-3’) |
| --- | --- | --- | --- |
| Mutation primers | | | |
| 291_AmpC_R1 Fw | Amplification of PAO1 *ampC* upstream region | PAO1 | ggaagcataaatgtaaagcaagcttccaatctctgctccaaatttttctaatggc |
| 292_AmpC_R1 Rv | Amplification of PAO1 *ampC* upstream region | PAO1 | ggcgagatagccgaacaggtcgatgctcgggttggaatagagg |
| 293_AmpC_R2 Fw | Amplification of PAO1 *ampC* downstream region | PAO1 | ctattccaacccgagcatcgacctgttcggctatctcgccg |
| 294_AmpC_R2 Rv | Amplification of PAO1 *ampC* downstream region | PAO1 | gagctcgagcccggggatccagccaggaccggcg |
| 295_AmpC_Check R1 | Checking of *ampC* mutation in PAO1 |  | cggcgagatagccgaacaggtc |
| 296_ AmpC_Check R2 | Checking of *ampC* mutation in PAO1 |  | gcctctattccaacccgagcatcgac |
| 297_ AmpD_R1 Fw | Amplification of PAO1 *ampD* upstream region | PAO1 | ggaagcataaatgtaaagcaagcttgaagctcagcaccagcgc |
| 298_ AmpD_R1 Rv | Amplification of PAO1 *ampD* upstream region | PAO1 | cccggaacgcatccagggctactgcgatatcgccccgg |
| 299_ AmpD_R2 Fw | Amplification of PAO1 *ampD* downstream region | PAO1 | tccggggcgatatcgcagtagccctggatgcgttccg |
| 300_ AmpD_R2 Rv | Amplification of PAO1 *ampD*  downstream region | PAO1 | aattcgagctcgagcccggggatcccgcctgctggacgatgc |
| 301_ AmpD_Check R1 | Checking of *ampD* mutation in PAO1 | PAO1 | ccggaacgcatccagggcta |
| 302_ AmpD_Check R2 | Checking of *ampD* mutation in PAO1 | PAO1 | gctccggggcgatatcgcagtag |
| 443a_pFOG-clonage_Fw | Amplification of pFOG plasmid by PCR | pFOG | GGATCCACTAGTTCTAGAGCGGCC |
| 444a_pFOG-clonage_Rv | Amplification of pFOG plasmid by PCR | pFOG | GAATTCGATATCAAGCTTATCGATACCGTCG |
| 473a_ampC 198-198_Fw | Amplification of PaS *ampC* gene | PaS | CCCAGTCTCGAGGTCGACGGTATCGATAAGCTTGATATCGAATTCatgcgcgataccagattcccc |
| 474a_ampC 198-198_Rv | Amplification of PaS *ampC* gene | PaS | CTGGAGCTCCACCGCGGTGGCGGCCGCTCTAGAACTAGTGGATCCtcagcgcttcagcggca |
| 475a_ampD 197-199_Fw | Amplification of PaS *ampD* gene | PaS | CCCAGTCTCGAGGTCGACGGTATCGATAAGCTTGATATCGAATTCgcctgctggacgatgcc |
| 476a_ampD 197-199_Rv | Amplification of PaS *ampD* gene | PaS | CTGGAGCTCCACCGCGGTGGCGGCCGCTCTAGAACTAGTGGATCCcgccgcctgttcgc |
|  | | | |
| qPCR primers | | | |
| 319_rpsL_Fw |  | *P. aeruginosa* | TATGCACCCGCGTATACACC |
| 320_rpsL_Rv |  | *P. aeruginosa* | TGTGACCTTCACCACCGATG |
| 315_ampC_Fw |  | *P. aeruginosa* | CCGTAGCCATCAGCCTGAAA |
| 316_ampC_Rv |  | *P. aeruginosa* | GTGAAGGTCTTGCTCACCGA |


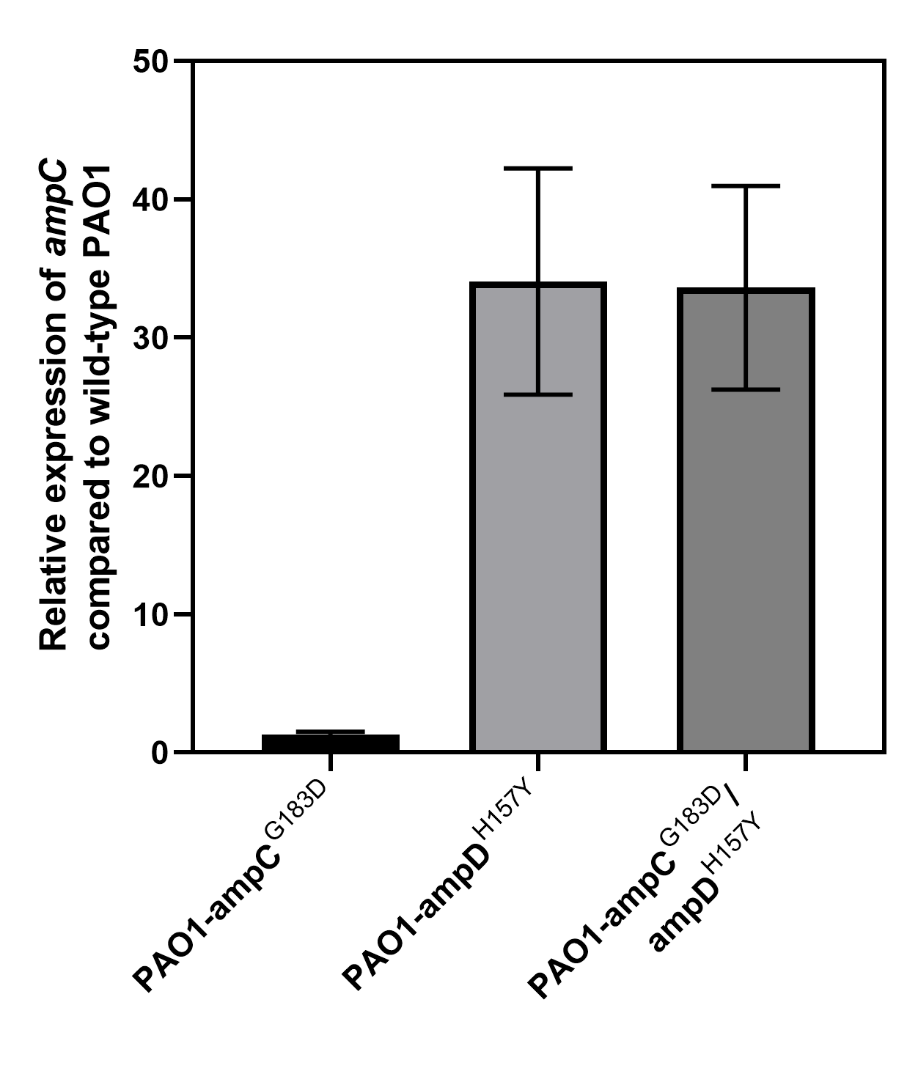


**Figure S1.** Relative expression of *ampC* gene in PAO1 mutant-derived strains compared to the reference wild-type PAO1. Mean values are represented with standard deviation (n=2).


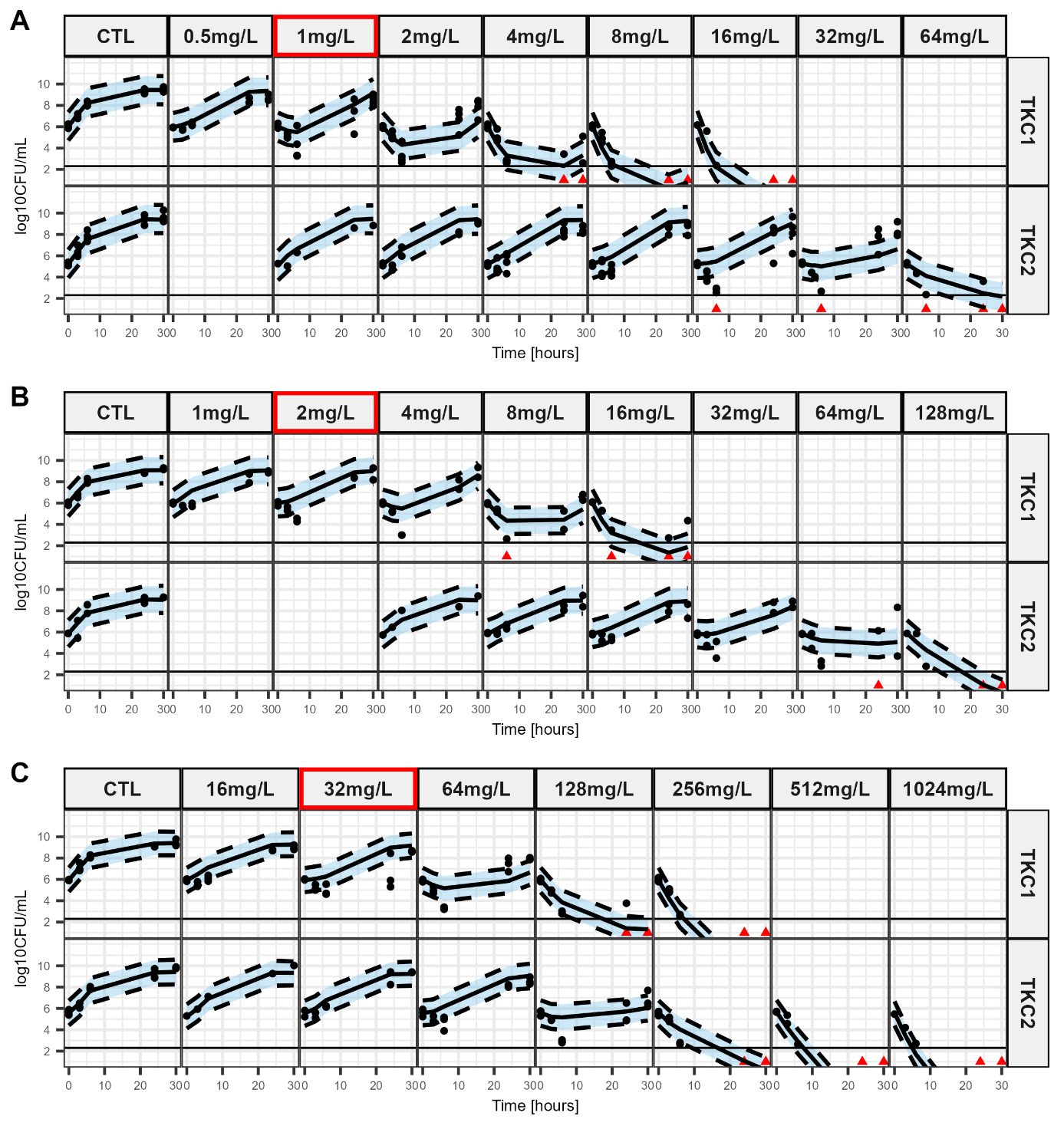


**Figure S2.** Visual predictive checks of the final model based on *in vitro* sequential time kill data ceftolozane/tazobactam for clinical and mutant-derived isolates. The plots show the observed time-kill data at different C/T concentrations with model predictions as medians and 80% confidence intervals around the median (shaded area). The red frame represents the MIC value of the corresponding strain. Dots represent countable plates; triangles represent data below the limit of quantification (BLQ: 2.3 log10 CFU/mL). Data below the limit of detection are plotted as 0.1 CFU/mL. A. PaS B. PaR AmpC^PaS^/AmpD^PaS^, C. PaR


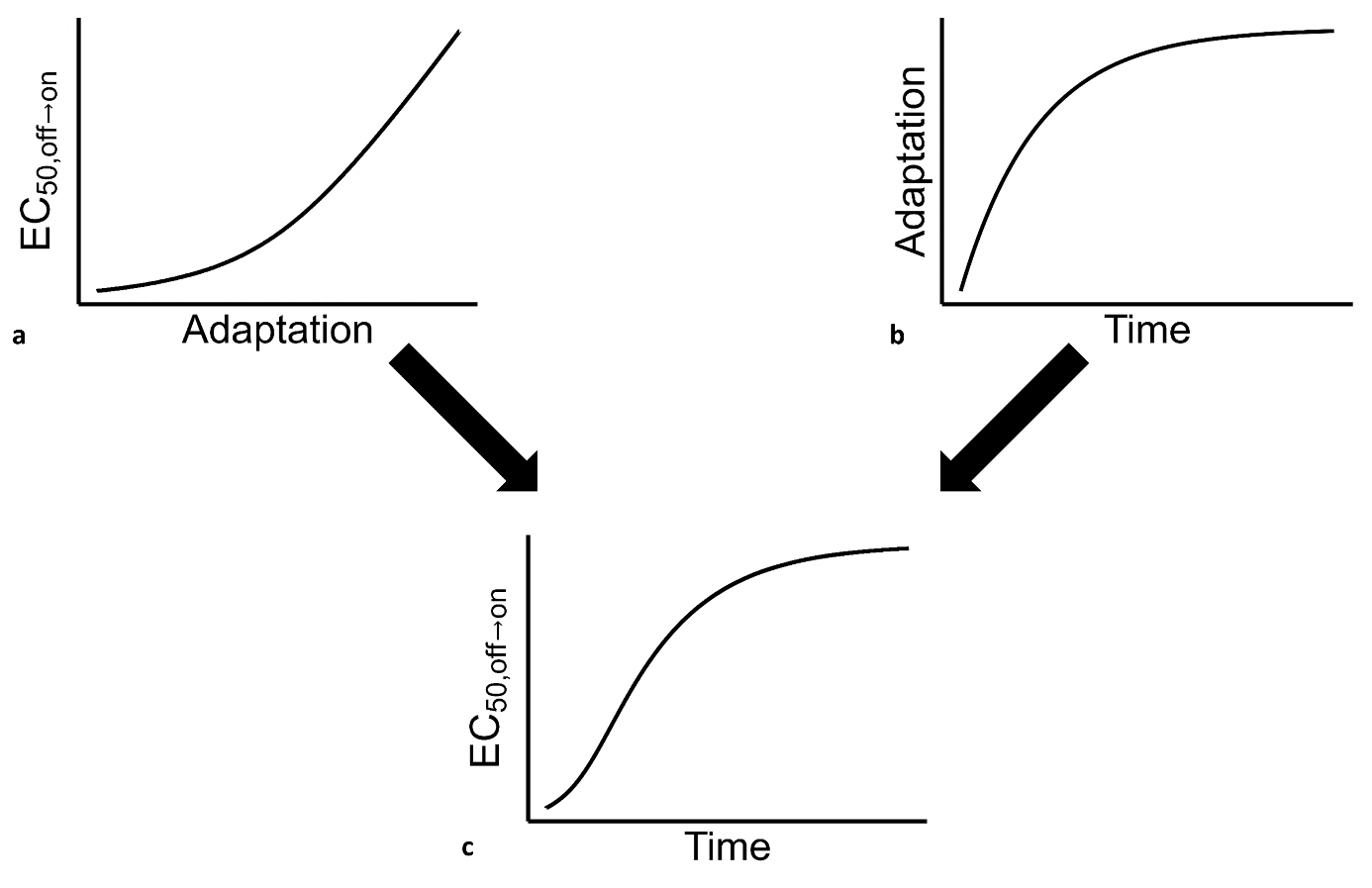


**Figure S3**. Schematic representation of indirect dependence of EC_50 off→on_ on time post antibiotic dosing. **a.** EC_50 off→on_ increases when the % of adapted bacteria increases. **b.** Also, the % of adapted bacteria increases over time. **c.** Thus, EC_50 off→on_ increases with time post antibiotic dosing.

Supplemental text S1: Estimation and evaluation methods

Bacterial count data were transformed into decimal logarithms before parameter estimation. For each manipulation and strain, the initial inoculum was standardized by the median of bacterial count at t=0 h.

All data were analyzed simultaneously using the non-linear mixed effect modelling approach in NONMEN 7.4 (ICON Development Solutions, Ellicott City, MD, USA). This approach allows simultaneous estimation of the average population parameters and inter-strain variability. All model estimations were conducted using Laplacian numerical algorithm. Dataset preparation was performed using R software. Pirana v2.9.2 was used to assist in model development.

Data below the LOQ were taken into account in the model estimation by applying Beal’s M3 method (1). The residual errors, the difference between the observed and model-predicted values at each time point, were additive on a log scale for bacteria counts (log10 CFU/mL).

Model selection was based on objective function value (OFV), goodness of fit plots (GOF) and uncertainty around population parameter estimates (relative standard errors RSEs). When two models were nested, a decrease in OFV of at least 3.84 (chi square 1df p = 0.05) was needed to select the most complex model (Likelihood ratio test LRT). For non-nested models, a decrease in the Akaike information criterion (AIC) and Bayesian information criterion (BIC) was needed to select of the best models. RSEs were obtained using the Sampling Importance Resampling (SIR) procedure (2) implemented in PsN.

Visual predictive checks (VPCs) based on 1000 simulations were drawn to evaluate the fit to data and taken into account for model selection.

Maximal observed effect (E_max_) and adaptation rate (k_on_) were close enough within the PAO1 strains group and the clinical strains group to be able to be estimated by one value per strain group. The other parameters were different for each strain.

Final model equations

$\frac{dBacteria}{dt}=k_{g}\times\left( 1-\frac{Bacteria}{B_{max}} \right)\times Delay\times Bacteria-Effect\times Bacteria$ , IC = 10^Inoc^ Suppl. eq. 2

$\frac{dAR_{off}}{dt}=k_{off}\times AR_{on}-k_{on}\times AR_{off}$ , IC=1 Suppl. eq. 3

$\frac{dAR_{on}}{dt}=k_{on}\times AR_{off}-k_{off}\times AR_{on}$ , IC=0 Suppl. eq. 4

$Effect= \frac{E_{max}\times C_{C/T}}{EC_{50, off}+C_{C/T}}\times AR_{off}+\frac{E_{max}\times C_{C/T}}{EC_{50,on}+C_{C/T}}\times AR_{on}$

Suppl eq. 5

$Delay=\frac{Time^{20}}{T_{lag}^{20}+ Time^{20}}$ Suppl eq. 6

Supplemental text S2: Antibiotic stability assays.

Analysis of IMI in the MHB was performed by an LC-MS/MS methods, previously described and developed for the quantification of IMI in rat plasma (3). Calibration curves were established in MHB II solution over 0.1 to 100 μg/mL. After collection, samples were precipitated by the addition of 200 µL acetonitrile and 25 µL of the internal standard at 50 µg/mL were added. Following the tubes were vortexed for 30 s and then centrifuged at 17,160×g for 5 min at 4 °C. One hundred eighty microliters (180 μL) of supernatant was transferred into a vial containing twenty microliters (20 μL) of mobile phase (see later). The system included a Waters high-performance liquid chromatography system module (Alliance 2695; Waters, Saint-Quentin en Yvelines, France) coupled with a Quattro micro Api mass spectrometer (Waters, Saint-Quentin en Yvelines, France). The compounds was analyzed on an XBridge Peptide BEH300 C18 column (5 μm, 2.1 x 150 mm, Waters). The mobile phase consisted of a mixture of water/ acetonitrile/formic acid /amomium formate 2.5mM (80%/20%/0.1%/2.5mM) was delivered isocratically at 0.18 mL/min. Electrospray ionization in both positive was used for the detection. Ions were analyzed in the multiple reaction monitoring, and the following transitions were inspected: m/z 300.2→98.0 and 300.2→142.0 for IMI , m/z 476.1→432.1 for ertapenem   (its internal standard). The intraday variability was characterized at three concentration levels (75, 5 and 0.4 μg/ml) a precision and bias of <7% for both compounds. Corresponding between-day variability was determined with a precision and a bias of <12% (n = 12).

Analysis of C/T in the MHB was performed by an LC-MS/MS methods, previously described and developed for the quantification of C/T in human plasma and urine (4). Calibration curves were established in MHB II solution over 0.1 to 100 μg/mL (ceftolozane) and 0.1 to 10 μg/mL (Tazobactam). After collection, samples were precipitated by the addition of 300 µL acetonitrile containing the internal standard at 2.5 µg/mL and 5 µg/mL for [15N2, 2H4]-ceftolozane and [13C2,15N3]-tazobactam respectively. Following the tubes were vortexed for 30 s and then centrifuged at 17,160×g for 5 min at 4 °C. One hundred eighty microliters (180 μL) of supernatant was transferred into a vial containing twenty microliters (20 μL) of 0.1% formic acid in water solution and transferred to an autosampler vial for analysis. The system included a Shimadzu high-performance liquid chromatography system module (Nexera XR; Shimadzu, Marne la Vallée, France) coupled with a TQ3500 mass spectrometer (Sciex, Les Ulis, France). The compounds was analyzed on a Kinetex® - F5 -100Å column (5 μm, 2.1 x 150 mm, Phenomenex). The mobile phase A consisted of water with 0.1% formic acid, and mobile phase B was acetonitrile with 0.1% formic acid. The gradient elution program started with 2% of mobile phase B, which was maintained for 1 min, then ramped to 95% mobile phase B by 6 min and that composition was maintained until 1 min. The mobile phase composition was then reverted to 2% mobile phase B at 8.1 min and that was maintained until the end of the chromatographic run at 11 min. Electrospray ionization in both positive and negative modes were used for the detection of ceftolozane and tazobactam, respectively. Ions were analyzed in the multiple reaction monitoring, and the following transitions were inspected: m/z 667.1→199.2 for ceftolozane, m/z 673.1→205.2 for its labeled internal standard, and m/z 299.0→67.9 for tazobactam and m/z 303.9→138.0 for the tazobactam labeled internal standard. The intraday variability was characterized at three concentration levels (75, 5 and 0.25 μg/mL for ceftolozane; 7.5, 5, 0.25 μg/mL for tazobactam) with a precision and bias of <15% for both compounds.)

**Supplemental references:**

(1) S L Beal. Ways to fit a PK model with some data below the quantification limit. J Pharmacokinet Pharmacodyn. 2001 Oct;28(5):481-504. doi: 10.1023/a:1012299115260.

(2) AG Dosne , M Bergstrand , MO Karlsson. An automated sampling importance resampling procedure for estimating parameter uncertainty. J Pharmacokinet Pharmacodyn. 2017 Dec;44(6):509-520.doi: 10.1007/s10928-017-9542-0.

(3) L Peng, X Wang, H Dang. Simultaneous determination of meropenem and imipenem in rat plasma by LC–MS/MS and its application to a pharmacokinetic study. Biomedical Chromatography. 2021;35:e5185. https://doi.org/10.1002/bmc.5185

(4) W.C Putnam, RR Kallem, V Edpuganti, I Subramaniyan, RG Hall. Development and validation of a quantitative LC-MS/MS method for the simultaneous determination of ceftolozane and tazobactam in human plasma and urine. Journal of Chromatography B. Volume 1159, 30 November 2020, 122354
